# Tissue-Dependent Consequences of *Apc* Inactivation on Proliferation and Differentiation of Ciliated Cell Progenitors via Wnt and Notch Signaling

**DOI:** 10.1371/journal.pone.0062215

**Published:** 2013-04-30

**Authors:** Aimin Li, Belinda Chan, Juan C. Felix, Yiming Xing, Min Li, Steven L. Brody, Zea Borok, Changgong Li, Parviz Minoo

**Affiliations:** 1 Division of Newborn Medicine, Department of Pediatrics, Los Angeles County+University of Southern California Medical Center, Keck School of Medicine of USC, Los Angeles, California, United States of America; 2 Department of Pathology, Los Angeles County+University of Southern California Medical Center, Los Angeles, California, United States of America; 3 Pulmonary and Critical Care Medicine, Department of Medicine, Washington University School of Medicine, St. Louis, Missouri, United States of America; 4 Will Rogers Institute Pulmonary Research Center, Division of Pulmonary and Critical Care Medicine, Department of Medicine, and Department of Biochemistry and Molecular Biology, University of Southern California, Keck School of Medicine of USC, Los Angeles, California, United States of America; 5 The State Key Laboratory for Agro-biotechnology, China Agricultural University, Beijing, China; Rush University Medical Center, United States of America

## Abstract

The molecular signals that control decisions regarding progenitor/stem cell proliferation versus differentiation are not fully understood. Differentiation of motile cilia from progenitor/stem cells may offer a simple tractable model to investigate this process. Wnt and Notch represent two key signaling pathways in progenitor/stem cell behavior in a number of tissues. Adenomatous Polyposis Coli, Apc is a negative regulator of the Wnt pathway and a well known multifunctional protein. Using the cre-LoxP system we inactivated the *Apc* locus via *Foxj1-cre*, which is expressed in cells committed to ciliated cell lineage. We then characterized the consequent phenotype in two select tissues that bear motile cilia, the lung and the testis. In the lung, *Apc* deletion induced β-catenin accumulation and Jag1 expression in ciliated cells and by lateral induction, triggered Notch signaling in adjacent Clara cells. In the bronchiolar epithelium, absence of *Ap*c blocked the differentiation of a subpopulation of cells committed to the ciliogenesis program. In the human pulmonary adenocarcinoma cells, Apc over-expression inhibited *Jag1* expression and promoted motile ciliogenic gene expression program including *Foxj1*, revealing the potential mechanism. In the testis, *Apc* inactivation induced β-catenin accumulation in the spermatogonia, but silenced Notch signaling and depleted spermatogonial stem cells, associated with reduced proliferation, resulting in male infertility. In sum, the present comparative analysis reveals the tissue-dependent consequences of *Apc* inactivation on proliferation and differentiation of ciliated cell progenitors by coordinating Wnt and Notch signaling.

## Introduction

Motile cilia perform many vital functions both during embryonic development and in maintenance of various organs. In early development, motile cilia are essential for establishment of embryonic left-right asymmetry. They are also necessary for normal lung function and fertility. Mutations causing ciliary deficiency underlie the human syndrome Primary Ciliary Dyskinesia (PCD) [1]. Emergence of fully differentiated ciliated cells from progenitor/stem cells is a tightly orchestrated step-by-step process that is amenable to detailed genetic and biochemical analysis. As such ciliogenesis may be exploited to address questions regarding the role of specific signaling pathways and how they impact progenitor/stem cell decision-making related to proliferation and differentiation under homeostatic conditions and in the face of injury, repair or remodeling.

The tumor suppressor Adenomatous Polyposis Coli, Apc is a key component of the destruction complex in the Wnt pathway that enables the maintenance of signaling within a homeostatic range. The function of the destruction complex relies on the ability of Apc to promote phosphorylation of β-catenin (Ctnnb1) via Gsk3b. In its inactive state, Gsk3b fails to phosphorylate Ctnnb1, leading to its cytoplasmic accumulation and subsequent transport to the nucleus where it affects transcription of several Wnt-target genes including *c-Myc and Axin2* [*2*]. Thus absence of Apc leads to stabilization of Ctnnb1 and activation of Wnt signaling. Apc has additional roles unrelated to regulation of Ctnnb1. It binds directly and indirectly to microtubules, regulating their assembly, cell migration, chromosomal stability and mitotic spindles [3]. Mutations in Apc affect cell proliferation, differentiation, apoptosis and migration. Low levels or absence of Apc is associated with the ability of cells to serve as functional tissue-embedded stem cells [4]. Previously, using the developing murine lung model, we found that Apc is expressed at high levels in airway ciliated cells [5]. The function of Apc in cells with motile cilia and whether its aberrations have a role in such disorders as PCD remain unexplored.

Studies in species from *Drosophila* to humans also implicate Notch signaling in establishment of asymmetry, cell fate decision and timing of differentiation during development. In the mouse lung, Notch receptor expression is seen in cells of endodermal as well as mesodermal origins [6]. Of the five Notch ligands, Jagged 1 is known to be induced by Wnt/Ctnnb1 signaling. Both receptors and ligands are heterodimeric type I membrane proteins that require cell–cell contact for activation. Ligand-receptor interactions trigger cleavage of the Notch intracellular domain (NICD) by gamma-secretase [6]. Thus detection of NICD has been an accepted marker of Notch pathway activation. Biological integration of Wnt and Notch signaling has been observed in the establishment and maintenance of diverse tissues and organs either collaboratively [7] or antagonistically [8].

The forkhead box transcription factor Foxj1 is specifically expressed in cell types that bear motile cilia in various organs including lung and testis, and drives the motile ciliogenesis program by directly stimulating the expression of various genes [9]. In the mouse lung, Foxj1 is initially detected in airway epithelial cells at E15.5, before the appearance of cilia, and thus is an early marker of ciliated cell fate determination [10]. In the mouse testes, Foxj1 expression is associated with the appearance of haploid germ cells and confined to specific stages of spermatogenesis [11].

Zhang et al used a *Foxj1-cre* line to mediate genomic recombination of floxed alleles specifically in cells committed to ciliary lineage [12]. Using the same *Foxj1-cre* driver line we generated mice carrying a targeted disruption of Apc specifically in ciliated cell progenitors. We subsequently analyzed and compared the phenotype of lungs and testes of the mutant mice. We found that absence of Apc caused expected hyperaccumulation of Ctnnb1 in both the airway epithelium and the spermatogonia. In the mutant lungs, *Foxj1-cre*-mediated inactivation of Apc triggered expression of Jag1, induced Notch signaling and blocked differentiation of a select group of bronchiolar ciliated cells. In contrast to the lung, absence of Apc silenced Notch signaling associated with progressive depletion of spermatogonial stem cells. We therefore propose that Apc may be crucial to ciliate cell differentiation and spermatogonial stem cell self-renewal via coordination of Wnt∼Notch cross-talk.

## Materials and Methods

### Mice

All animals were maintained and housed in pathogen-free conditions according to a protocol approved by The University of Southern California Institutional Animal Care and Use Committee (IACUC) (Los Angeles, CA, USA).*The Apc^flox/flox^* mice and *Foxj1-cre* transgenic mice were kindly provided by Dr. Raju Kucherlapati (Harvard Medical School, Boston, MA) [13] and Dr. Michael J. Holtzman (Washington University School of Medicine, St. Louis, MO) [12], respectively. Ciliated cell progenitor specific Apc conditional knockout mice, *Apc^Foxj1^* (*Apc^flox/flox^; Foxj1-cre*) were created by crossing *Apc^flox/flox^* homozygous females with *Apc ^flox/wt^; Foxj1-cre* double heterozygous males and genotyped as previously described [14].

*Axin2-LacZ* and *TopGal-lacZ* Wnt activity reporter mouse lines were described previously [15]. Triple transgenic *Foxj1-cre; Apc^flox/flox^; Axin2-LacZ* (simply *Apc^Foxj1^; Axin2*) and *Foxj1-cre; Apc^flox/flox^; TopGal (*simply *Apc^Foxj1^; TopGal*) mice were generated and LacZ activity was assessed by X-gal staining or by immunohistochemistry as previously reported [16].

### Naphthalene Exposure

Adult female *Apc^flox/flox^* (*Control)* and *Apc^Foxj1^* mice were injected with 300 mg/kg Naphthalene (Sigma) dissolved in Mazola corn oil (Cordova, TN). Animals were sacrificed at 1 day and 7 days following Naphthalene exposure.

### LacZ (β-galactosidase) Staining

Activity of β-galactosidase in lungs and testis was determined by X-gal staining as described previously [15].

### Western Blot

Protein extract preparation and western blot analysis were carried out as previously described [17]. Primary antibodies used are described in Table S1.

### Immunohistochemistry (IHC)

Tissue slides were deparaffinized and rehydrated through an alcohol gradient series to water. Antigens were retrieved and endogenous peroxidase activity was quenched using 3% hydrogen peroxide. After normal serum blocking, the sections were incubated with primary antibodies at 4°C overnight. Impress-anti-rabbit or anti-mouse IgG (Vector Laboratories) was applied for 50 min at room temperature. Staining was visualized by Peroxidase Substrate Kit DAB (Vector Laboratories).

For immunofluorescence (IF) staining, the sections were incubated with primary antibodies overnight at 4°C and then reacted with a mixture of Cy3 anti-rabbit IgG or fluorescein anti-mouse or anti-goat IgG (Jackson ImmunoResearch Laboratories, INC) for 1 hr in the dark at RT. After thorough rinses with PBS containing 0.1% Triton X-100, the sections were mounted with VECTASHELD mounting medium containing DAPI to visualize nuclei. Primary antibodies used are described in Table S1. Stained sections were visualized on a Zeiss fluorescent microscope and images captured with a SPOT INSIGHT QE camera (Diagnostic Instruments) and analyzed using SPOT Advanced software (Diagnostic Instruments).

### Morphometric Analyses

The percentage of cells immunostained for Ki67, βIV-tubulin and Ctnnb1 was determined by counting cells in lung or testis sections from control and mutant animals at 40X magnification. For each maker, ten fields or a minimum of 1000 cells were analyzed in at least three animals per group. Data were represented as mean±standard deviation (SD). Statistical analysis was performed using Student's *t*-test. Differences were considered significant at *P*<0.05.

### Real Time Polymerase Chain Reaction (PCR)

DNase-free RNA was prepared using Trizol reagent (Invitrogen) according to the manufacturer’s instructions. After DNase treatment, RNA was reverse-transcribed to cDNA using the Superscript III kit (Invitrogen) according to the manufacturer’s instructions. The cDNA was subjected to real-time PCR using SYBR Green PCR Master Mix with a LightCycler (Roche Applied Sciences, IN) as previously described [18]. Gene expression was normalized to β-actin or TBP define. All primers for real time PCR were designed by using the program of Universal Probe Library Assay Design Center from Roche Applied Sciences (IN).

### Cell Culture and Transient Transfection Assays

Human pulmonary adenocarcinoma H441 cells (ATCC) were used for transfection experiments. Cells were maintained in RPMI 1640 containing 10% fetal bovine serum and 1% penicillin-streptomycin (Invitrogen Life Technologies, Inc). Transfection of Apc expression constructs to H441 cells was performed with SuperFect (Qiagen) as described previously [14]. Twenty-four hours after transfection, cells were harvested and analyzed for gene expression by real time PCR.

## Results

### *Foxj1-cre*-mediated Recombination in the Lung and Testis

In whole mount preparations, LacZ activity in *Foxj1-cre; Rosa26* lungs was clearly detectable only after embryonic day E18.5 but persisted in postnatal samples (Fig. S1, A–C and G–I). Using an anti-β-galactosidase (β-gal) antibody was more sensitive, identifying cre activity in tracheal, bronchial and bronchiolar epithelium of E16.5 lungs (Fig. S1, D–F). Foxj1-cre activity pattern in later stages of lung development (4–6 weeks of age) has been previously reported (Zhang et al., 2007). In the testes, *Foxj1-Cre*-mediated recombination activity in 4–6 week old mice was found primarily in the spermatogonial stem cells (Zhang et al., 2007). In the present study using *Foxj1-cre; mT/mG* double transgenic older mice (>2 months of age) we observed green fluorescence not only in the spermatogonia, but also in their descendents, the spermatids and spermatozoa (Fig. S2), which is consistent with endogenous expression pattern of *Foxj1* in the testis [10].

### *Foxj1-cre-*mediated Deletion of *Apc* Induces Ctnnb1 Accumulation

**Figure 1:**
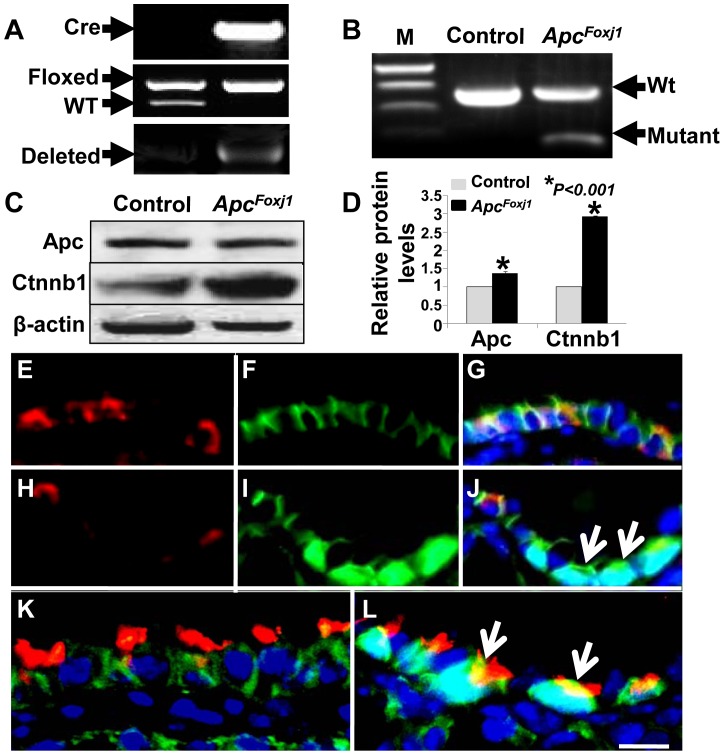
*Foxj1-cre*-mediated ciliate cell progenitor-specific deletion of *Apc*. **A** Deletion of *Apc* exon 14 was validated by PCR with genomic DNA. Top panel shows a band of 370 bp for Foxj1-cre. Middle panel shows bands of 320 bp for Wt and 430 bp for floxed product. Bottom panel shows a 500 bp fragment in mutant but not control DNA. **B** RT-PCR with primers spanning exon 14 using total RNA from control and *Apc^Foxj1^* lungs. Expected 528 bp (control) was detected in both control and *Apc^Foxj1^*lungs. A 313 bp (truncated Apc allele) was amplified only from *Apc^Foxj1^* lungs. **C & D** Western blot (**C)** and densitometric quantification (**D**) of total protein from control and *Apc^Foxj1^* lungs. β-actin was used as control. *P<0.01.**E–J** Immunostaining of Apc (red) and Ctnnb1 (green) in postnatal control (**E–G**) and *Apc^Foxj1^* (**H–J**) lungs. Arrows in **J** show selective accumulation of Ctnnb1 in cells without Apc. **K & L** Immunostaining of βIV-tubulin (red) and Ctnnb1 (green) in postnatal control (**K**) and *Apc^Foxj1^* (**L**) lungs. Arrows in **L** show accumulation of Ctnnb1 in βIV-tubulin^pos^ ciliated cells. Scale bar: 20 µm.

*Apc^flox/flox^* females were mated to mice with *Apc^flox/+^; Foxj1-cre* double heterozygous males to generate *Apc^flox/flox^; Foxj1-cre* homozygous mice (heretofore *Apc^Foxj1^*). *Apc^flox/flox^* mice carry a floxed allele of Apc exon 14, which upon excision causes a frameshift, thereby generating a truncated *Apc* that no longer binds Ctnnb1. To validate *Foxj1-cre*-induced recombination, DNA was amplified from tails or lungs by PCR using wild-type, or exon 14-specific primer sets as previously reported [13]. A PCR product showing deletion of exon 14 was amplified from *Apc^Foxj1^* lung DNA, but not from control lungs (Fig. 1A). Furthermore, analysis of *Apc* transcripts by RT-PCR, using primers spanning exon 14 revealed the expected 313 bp product from the truncated *Apc* allele only in the *Apc^Foxj1^* lungs (Fig. 1B). Western blot analysis showed significant increase in Ctnnb1 in *Apc^Foxj1^* lungs (Fig. 1C&D). IHC revealed that APC is absent specifically in cells with robust Ctnnb1 accumulation (Fig. 1E–J) and Ctnnb1 accumulation occurred specifically in βIV-tub^positive^ ciliated epithelial cells (Fig. 1K&L).

To determine whether the accumulated Ctnnb1 in the pulmonary ciliated cells was functionally active, we generated triple transgenic *Foxj1-cre; Apc^flox/flox^; Axin2-LacZ* (simply *Apc^Foxj1^*; *Axin2*). In these mice LacZ activity was increased specifically in cells with Ctnnb1 accumulation (Fig. S3). IHC further validated the LacZ-based observation. Axin2 was increased in cells with accumulated Ctnnb1 and was absent from Clara cells that are CC10^positive^ (Fig. S4). Thus the accumulated Ctnnb1 is functional. These data indicate that deletion of Apc activates the Wnt/Ctnnb1 signaling in the *Foxj1-cre^ positive^* lung epithelial cells. Despite this finding, Ki67 staining revealed a lower mitotic index for cells with accumulated Ctnnb1 compared to controls (Fig. S5). Analysis of apoptosis by TUNEL revealed no significant differences (Data not shown). These data are consistent with reports that Wnt/Ctnnb1 signaling has a limited role in cell proliferation and apoptosis in airway epithelium [19].

### Loss of Apc Blocks Ciliary Differentiation Only in a Subset of Bronchiolar Epithelial Cells

**Figure 2:**
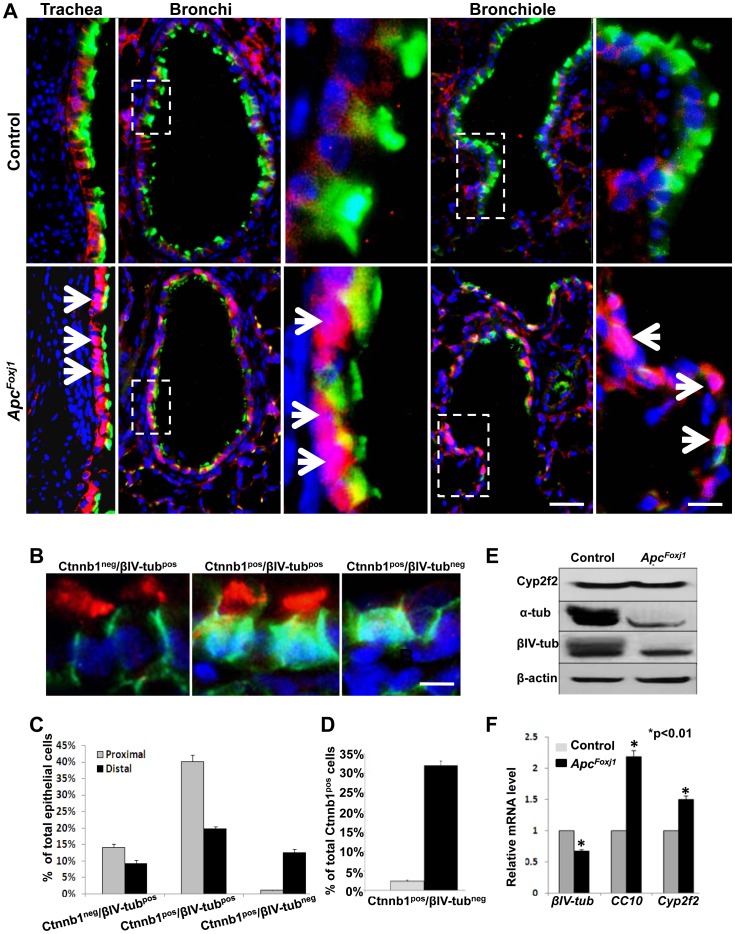
Loss of *Apc* blocks ciliary differentiation in a subset of bronchiolar epithelial cells. **A** Representative immunostaining images of βIV-tubulin (βIV-tub, green) and Ctnnb1 (red) in control and *Apc^Foxj1^ l*ungs at postnatal 2 weeks are shown. Arrows indicate accumulated Ctnnb1 signals. Scale bars are 20 and 10 µm, respectively. **B** Three groups of ciliate cells presented in the *Apc^Foxj1^* mice airway epithelium were identified by immunostaining of βIV-tub (red) and Ctnnb1 (green). Scale bar: 5 µm. **C** Quantification by manual counting of ratio of these cells vs total epithelial cells in the lungs from 2 week- to 2 month-old *Apc^Foxj1^* (n = 3) animals. **D** Quantification of ratio of Ctnnb1^pos^/βIV-tub^neg^ cells vs total Ctnnb1 accumulated cells in bronchi (proximal) and distal bronchiolar epithelium (distal) from 2 week- to 2 month-old *Apc^Foxj1^* (n = 3) animals. **E** Representative western blot analysis of total protein from adult control and *Apc^Foxj1^* lungs. **F** Relative quantification of fold induction or inhibition, compared to controls (arbitrarily adjusted to 1) of mRNA levels by real-time PCR (n = 3 for each genotype).

To elucidate the consequences of *Foxj1-cre*-mediated loss of Apc function on airway epithelial cell differentiation, we analyzed the expression of βIV-tubulin (βIV-tub), a ciliated cell marker by IHC on specific compartments of the conducting airway epithelium including trachea, bronchi and bronchioles. Results revealed that *Apc* inactivation and consequent accumulation of Ctnnb1 did not block cilia formation in the trachea and bronchi (Fig. 2A). This was in sharp contrast to the bronchiolar epithelium where clear inhibition of cilia formation was apparent (Fig. 2A). Indeed, three distinct subpopulations were identified. The first included cells distinguished by Ctnnb1^neg/^βIV-tub^pos^ phenotype. These are ciliated cells in which recombination failed (Ctnnb1^neg^), and ciliogenesis was normal (βIV-tub^pos^). The second group was Ctnnb1^pos^/βIV-tub^pos^ cells in which recombination occurred, but did not block ciliogenesis. And, finally the third group, distinguished by Ctnnb1^pos^/βIV-tub^neg^ profile. In these cells, commitment to ciliary differentiation occurred (Ctnnb1^pos^), but ciliogenesis was blocked (Fig. 2B). Manual counting of multiple samples revealed that in the proximal domain only a small percentage (1.3%) of the total number of airway epithelial cells was identified as Ctnnb1^pos^/βIV-tub^neg^. In contrast, the percentage of Ctnnb1^pos^/βIV-tub^neg^ airway epithelial cells in the more distal compartments was nearly an order of magnitude higher (11.3%) (Fig. 2C). The ratio of Ctnnb1^pos^/βIV-tub^neg^ cells over the total number of Ctnnb1^pos^ cells was further analyzed which revealed 2.4% Ctnnb1^pos^/βIV-tub^neg^ cells in proximal domain compared to 32% in distal domain (Fig. 2D). We then analyzed airway epithelial cell differentiation by Western blots and real time PCR using total lung protein or mRNA from *Apc^Foxj1^* & control mice. Western blot analysis revealed dramatic reduction of both βIV-tubulin and α-tubulin proteins in *Apc^Foxj1^* lungs (Fig. 2E). PCR analysis revealed that *βIV-tub* mRNA was reduced in mutant lungs compared to controls (Fig. 2F, 0.67±0.03, p<0.01). Interestingly, *CC10* & *Cyp2f2* RNAs, both expressed by Clara cells were increased in mutant lungs compared to controls (Fig. 2F, 2.19±0.12 vs.1 and 1.5±0.08 vs.1, respectively, p<0.01). Therefore, inactivation of *Apc* and consequent Ctnnb1 accumulation blocked cilia formation only in a distinct subpopulation of Foxj1 expressing bronchiolar epithelial cells.

### Loss of Apc Induces Notch Signaling in Bronchiolar Epithelium

**Figure 3:**
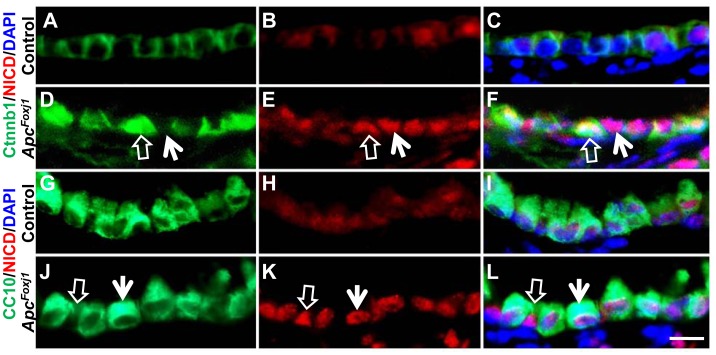
Loss of *Apc* induced Notch1 activation in bronchiolar epithelium of *Apc^Foxj1^* mice. Representative immunostaining of Ctnnb1 or CC10 (green) and cleaved Notch1 N–terminal intracellular domain (NICD) (red) in control (A–C and G–I) and *Apc^Foxj1^* (D–F and J–L) lungs at postnatal 2 week-old. Empty arrows in D–F indicate co-localization of Ctnnb1 and NICD^pos^ cells. Arrows in D–F indicate NICD^pos^ cells in non-Ctnnb1cells. Empty arrows in J–L indicate NICD^pos^ cells in CC10*^Neg^ cells*. Arrows in J–L indicate co-localization of CC10 and NICD. Scale bar: 10 µm.

Notch signaling plays an important role in controlling the number of ciliated versus secretory cells during airway epithelial cell differentiation [20]. Examination by IHC revealed low level of NICD in control lung epithelial cells (Fig. 3B). This contrasted with striking activation of Notch signaling as evidenced by nuclear NICD presence in a vast number of airway epithelial cells in the mutant lungs (Fig. 3E). Notch activation occurred in all luminal cells, but was particularly strong in those without accumulated Ctnnb1 (Fig. 3F) which were further identified as Clara cells (Fig. 3L).

**Figure 4:**
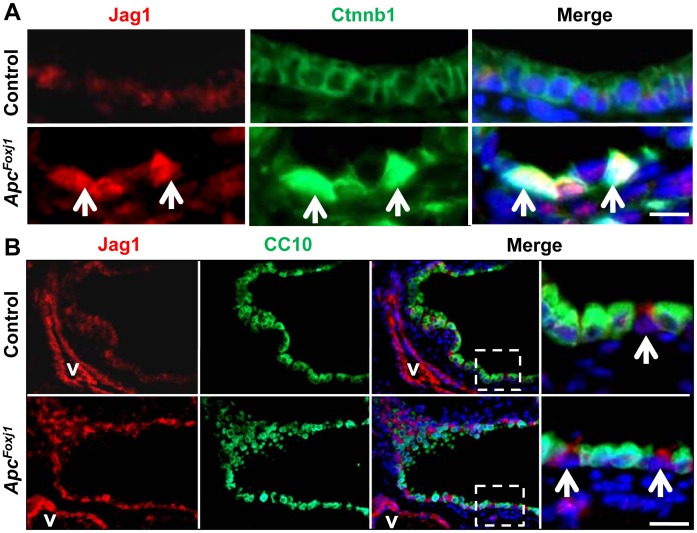
Loss of *Apc* induced Jag1 *expression* in bronchiolar epithelium of *Apc^Foxj1^* mice. **A** Representative immunostaining of Jag1 (red) and Ctnnb1 (green) in control and *Apc^Foxj1^* lungs at postnatal 2 week-old. Arrows show co-localization of Jag1 and Ctnnb1. Scale bar: 10 µm. **B** Representative immunostaining of Jag1 (red) and CC10 (green) in control and *Apc^Foxj1^* lungs at postnatal 2 week-old. Arrows show mutually exclusive localization of Jag1 and CC10. V, vasculature. Scale bar: 20 µm.

**Figure 5:**
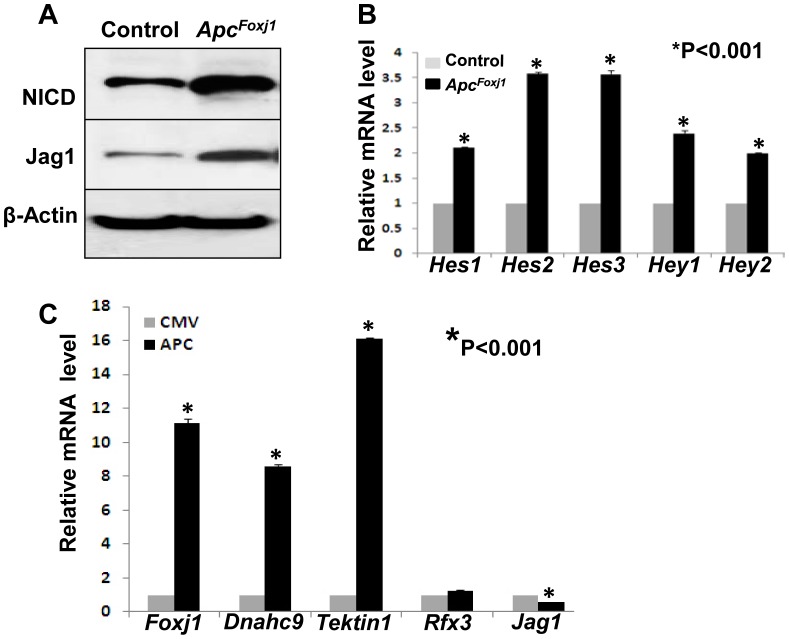
Loss of *Apc* induced Notch signaling in *Apc^Foxj1^* mice lung. **A** Representative western blots analysis of total protein from adult control and *Apc^Foxj1^* lungs. β-actin was used as control. **B** Real-time PCR of Notch-target genes in control and *Apc^Foxj1^* lungs from 2 week-old to adult. Values are fold induction or inhibition compared to c*ontrols* (arbitrarily adjusted to 1). (n = 3 for each genotype). **C** Real-time PCR of indicated mRNA levels in Human H441 pulmonary adenocarcinoma epithelial cells transfected with Apc expression construct (APC) or with vector alone (CMV) as a control. Values are fold induction or inhibition compared to c*ontrols* (arbitrarily adjusted to 1). Transfections were done in triplicate, and the mean ± SD are shown.

To determine the link between Apc inactivation and Notch activation, we examined the expression of Jag1, a Notch ligand that is expressed in the lung epithelium [20]. Significantly increased Jag1 was observed in cells with accumulated Ctnnb1 (Fig. 4A). Increased Jag1 was not found in Clara cells (Fig. 4B). Western blot (Fig. 5A) analysis validated increased Jag1 and NICD. Quantitative RT-PCR revealed increased mRNA for Hes and Hey, Notch signaling-triggered target genes (Fig. 5B). Thus, inactivation of Apc and consequent Ctnnb1/Wnt signaling increased Jag1 in Foxj1-expressing Ctnnb1^pos^ cells and activated Notch in adjacent Clara cells.

### Apc Inhibits Jag1 in Human Pulmonary Adenocarcinoma Epithelial Cells

To understand the mechanism whereby Apc may regulate ciliated cell differentiation and its relationship to Notch signaling, we used an in vitro cell culture model. Using H441 human pulmonary adenocarcinoma cell line, we found that overexpression of *Apc* decreased *Jag1* mRNA to near half of controls (Fig. 5C, 0.56±0.02 vs. 1, *p<*0.001), indicating that Apc can regulate Notch signaling via modulation of its ligand. Over-expression of Apc also increased *Foxj1* mRNA by 11.1-fold compared to controls (Fig. 5C). The latter data were further validated by the finding that two *Foxj1* downstream target genes, *Tektin* and *Dnahc9* [21] were also increased in transfected H441 cells by 16.2 and 8.6 fold respectively, while over-expression of Apc had no significant effect on expression of *Rfx3*, another cilia transcription factor, reportedly to be essential for the differentiation of nodal monocilia [22]. Together, these data strongly indicate that Apc acts upstream of Notch to promote ciliated cell fate determination and differentiation by regulating Foxj1 transcription factor in the airway epithelial cells.

### Accelerated Repair of Injured Airway Epithelium in *Apc^Foxj1^* Mouse Lungs

**Figure 6:**
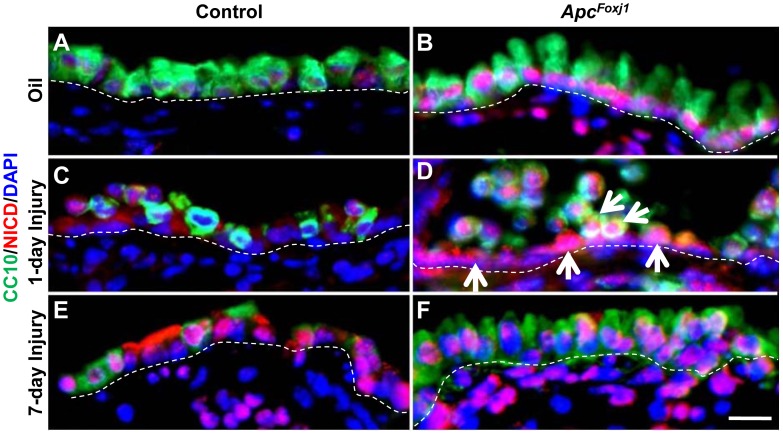
Accelerated regeneration of Clara cells in *Apc^Foxj1^* mouse lungs. Representative immunostaining of CC10 (green) and NICD (red) on adult lungs one and seven-days after Naphthalene treatment isolated from control and *Apc^Foxj1^* mice. Arrows in **D** point to NICD^pos^ cells. Note: Notch signaling was greatly activated as early as 1-day after Naphthalene injury and appeared in both necrotic CC10^pos^ Clara cells and squamous ciliate cells spread beneath injured Clara cells in the airway epithelium of mutant mice (**D**)**.** Scale bar: 10 µm.

Exposure of the bronchiolar epithelium to Naphthalene results in severe and rapid loss of the majority of Clara cells within 24 hours [5]. A select but small subpopulation of Clara cells are resistant to naphthalene and are thought to act as facultative progenitors during regeneration of the bronchiolar epithelium [23], [24]. We asked whether increased CC10 in *Apc^Foxj1^* lungs (Fig. 2F) indicated an expanded pool of facultative progenitor cells? To address this, naphthalene was administered to *Apc^Foxj1^* and control adult mice. Lung samples were processed on day 1 and 7. Within the first 24 hours, loss of Clara cells occurred in both the wild type and the mutant airways (Fig. 6C&D). Strong NICD was detected in the remaining cells in *Apc^Foxj1^* airways while a more subtle increase occurred in the controls (Fig. 6C&D). In mutant lungs, as early as day 7, the airways showed significant signs of Clara cell regeneration (Fig. 6F). This is contrasted with control lungs in which little regeneration was evident, although there was clear evidence for activation of Notch signaling (Fig. 6E). These data suggest that *Foxj1*-cre-mediated inactivation of *Apc* and hyperactivation of Ctnnb1/Wnt signaling accelerates the ability of facultative progenitor cells to repair the naphthalene-injured airway epithelium.

### Loss of *Apc* Depletes Germ Cells & Blocks Spermatogenesis

**Figure 7:**
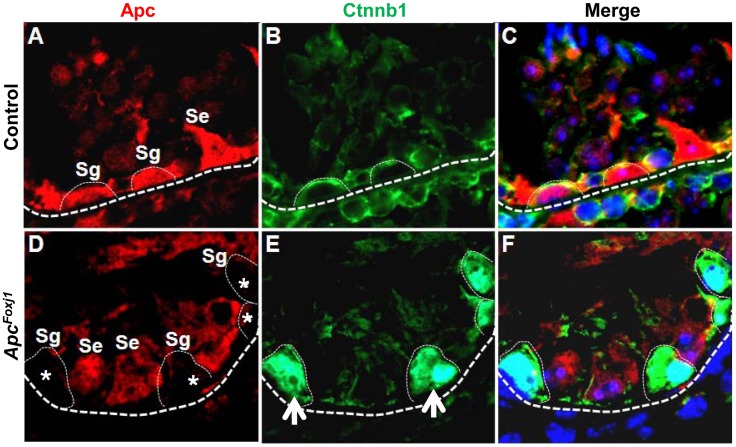
Loss of *Apc* results in selective accumulation of Ctnnb1 in the spermatogonia of adult *Apc^Foxj1^* testis. Representative immunostaining of APC (red) and Ctnnb1 (green) in control and *Apc^Foxj1^* testis at 12 months are shown. Asterisks in **D** show cells with absent Apc. Arrows in **E** show cells lacking Apc had Ctnnb1 accumulation. Sg: Spermatogonia; Se: Sertoli cells. Scale bar: 10 µm.

Male *Apc^Foxj1^* mice are infertile, suggesting changes in the testes, another site of *Foxj1* expression. In the control testes, Apc and Ctnnb1 were co-localized primarily to the cells adjacent to the outer membrane of the seminiferous tubule, the spermatogonia and the Sertoli cells (Fig. 7A–C). In the mutant testis, robust nuclear Ctnnb1 accumulation occurred in spermatogonia where Apc is undetectable (Fig. 7D–F). Again, significant increase of LacZ activity was seen in the testes of *Apc^Foxj1^*; *Axin2* mice (Fig. S6A) indicating accumulated Ctnnb1 is in fact functional in triggering the Wnt pathway. Specificity of this reaction was further validated by IHC showing colocalization of Ctnnb1 and Axin2 (Fig. S6C).

**Figure 8:**
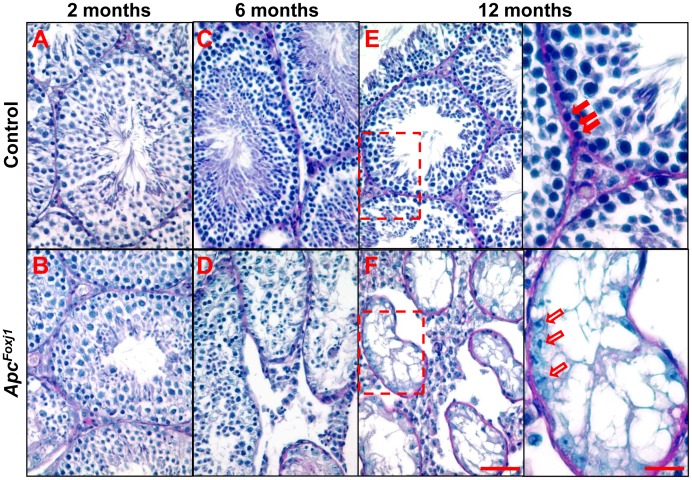
Loss of *Apc* results in progressive depletion of germ cells in the adult *Apc^Foxj1^* testis. PAS staining of various stages of testes from control littermate (A,C,E) and *Apc^Foxj1^* mice (B,D,F) are shown. Arrows in boxed area of **E** show spermatogonia on the basement membrane. Empty arrows in boxed area of **F** show absence of spermatogonia. Scale bars are 50 and 20 µm, respectively.

Analysis of testicular development and morphology in *Apc^Foxj1^* male mice showed that at earlier stages of development, from embryonic to 4 weeks of age, the testes of wild-type and *Apc^Foxj1^* mice were similar in size (data not shown). By 2 months of age, the *Apc^Foxj1^* testes consistently weighed about 40% less and by 6 months, 70% less than controls (Fig. S6A). In *Apc^Foxj1^* testes, PAS staining revealed a progressive loss of spermatogonia (Fig. 8). At 2 months of age, the testicular cross-sections of the *Apc^Foxj1^* mice showed germ cells from all stages of spermatogenesis although frequent loss of mature flagellated spermatozoa was readily seen (Fig. 8B). By 6 months, many *Apc^Foxj1^* testicular tubules had undergone progressive degeneration and spermatogonia, spermatocytes and spermatids were depleted significantly and mature spermatozoa were never seen (Fig. 8D). At 12 months, depletion of germ cells resulted in Sertoli cell-only seminiferous tubules (Fig. 8F).

**Figure 9:**
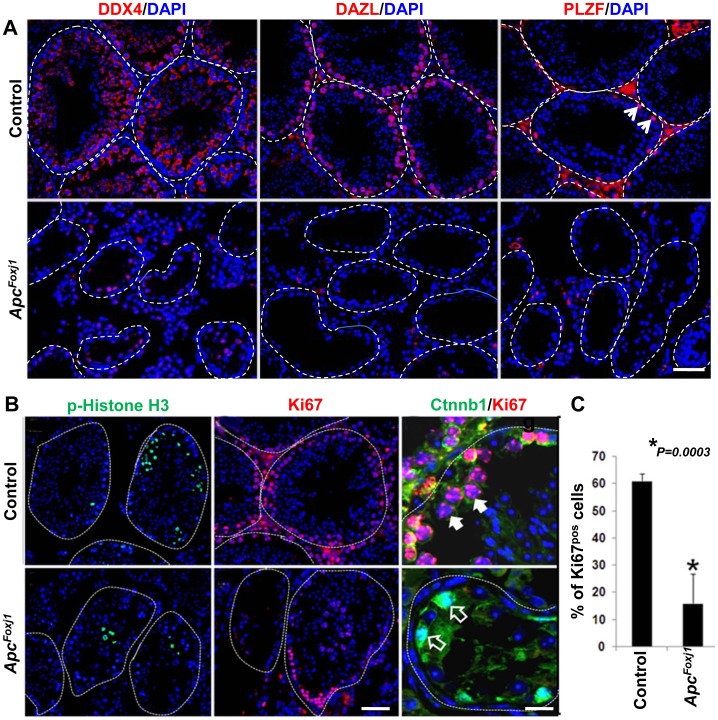
Loss of *Apc* causes spermatogonial stem cell (SSCs) depletion and blocks proliferation of SSCs in adult *Apc^Foxj1^* testes. **A** Representative immunostaining of DDX4, DAZL and PLZF in control and *Apc^Foxj1^* testis at 12 months are shown. Dotted lines indicate the basement membrane of seminiferous tubules. Arrows show PLZF^pos^ cells on the basement membrane. Scale bar: 50 µm. **B** Mitotic activity of germ cells in the adult control and *Apc^Foxj1^* testis. Representative immunostaining of Phospho-Histone-H3 (green), Ki67 (red) and double staining of Ki67 (red) and Ctnnb1 (green) in control and *Apc^Foxj^* testis at 6 or 12 month-old are shown. Note obvious decrease of Phospho-Histone-H3 or Ki67 staining in *Apc^Foxj1^* testis. Arrows show Ki67^pos^ cells; empty arrows show cells with *Ctnnb1^pos^* are not Ki67^pos^. Scale bars are 50 and 20 µm, respectively. **C** Manual counting of Ki67^pos^ cells in control and *Apc^Foxj1^* testis from 6 month to 12-month old (n = 4 for each genotype).

To verify the identity of the germ cells that remained in the seminiferous tubules of the mutant mice, three known germ cell markers, namely DDX4 [25], which is expressed in differentiated germ cells from spermatogonia to round spermatids; DAZL, expressed in both differentiated and undifferentiated spermatogonia and PLZF, a spermatogonial stem cell (SSC)-specific marker [26] were analyzed by immunofluorescence (Fig. 9A). In the control testes, DDX4-positive cells were present across the width of entire seminiferous epithelium; DAZL^positive^ cells were arranged at the periphery and PLZF^positive^ cells were present as a relatively small population. In sharp contrast, in the *Apc^Foxj1^* testes all three germ cell-specific makers were nearly undetectable. In the wild type testes Apc and PLZF were co-expressed in the gonocytes at postnatal day 3 and later in the SSCs (Fig. S7). Moreover, *Apc^Foxj1^* testes remarkably phenocopied PLZF null testes [26], [27]. Thus it appears that similar to PLZF, Apc may be necessary for self renewal of SSCs.

Germ cell depletion could result from alterations in spermatogonial proliferation or apoptosis. IHC revealed that Ki67^positive^ cells were arranged uniformly at the periphery of seminiferous tubules of control mice (Fig. 9B). On the contrary, Ki67^positive^ cells were either entirely absent or found in clusters in *Apc^Foxj1^* mice. In addition, cells with accumulated Ctnnb1 were not Ki67^positive^ (Fig. 9B, empty arrows). This is consistent with the findings in the lung epithelium (Fig. S5A). Phospho-Histone-H3 staining further confirmed decreased mitotic activity in the mutant testes (Fig. 9B). Manual cell counts revealed Ki67^positive^ cells were significantly reduced in *Apc^Foxj1^* testes (Fig. 9C). TUNEL assessment showed no differences (data not shown). Thus, absence of Apc blocks proliferation of SSCs and causes spermatogonial depletion.

### Loss of *Apc* Inhibits *Notch* Signaling in Testicular Epithelium

**Figure 10:**
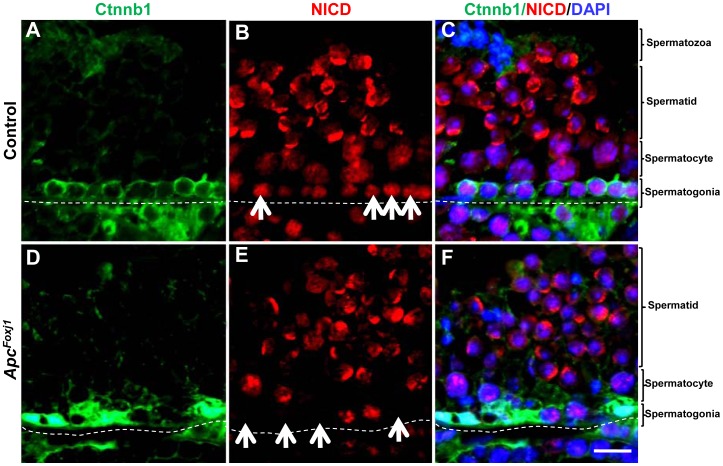
Loss of *Apc* inhibits Notch1 activation in *Apc^Foxj1^* mouse testis. Representative immunostaining of Ctnnb1 (green) and NICD (red) in control and *Apc^Foxj1^* mouse testes at 2 months are shown. Arrows in B indicate NICD^pos^ cells. Arrows in E indicate disappearance of NICD^pos^ cells in Ctnnb1 accumulated cells. Scale bar: 20 µm.

**Figure 11:**
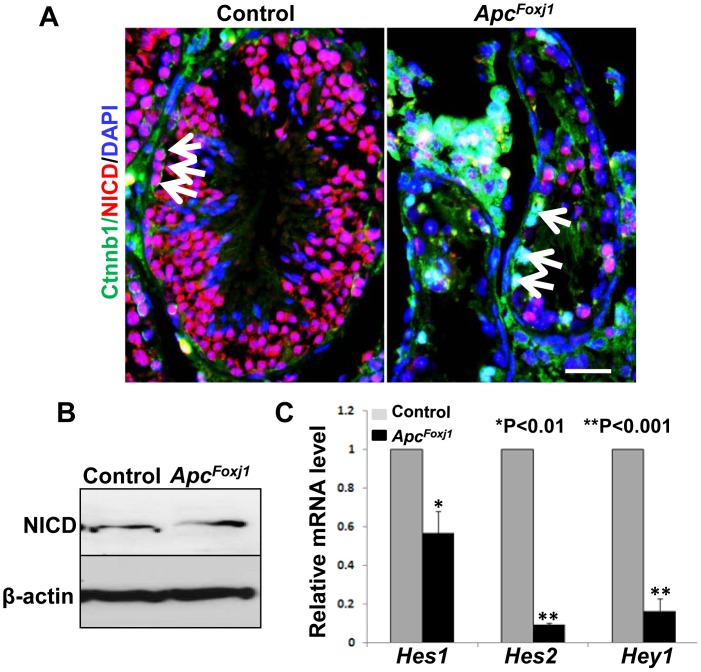
Loss of *Apc* inhibits Notch signaling in *Apc^Foxj1^* mouse testis. **A** Representative immunostaining of Ctnnb1 (green) and NICD (red) in control and *Apc^Foxj1^* testes at 12 months are shown. Arrows in control mouse testis indicate NICD^pos^ spermatogonia stem cells. Arrows in ***Apc^Foxj1^*** mouse testis indicate disappearance of NICD^pos^ signals in Ctnnb1 accumulating spermatogonia. Scale bar: 30 µm. **B** Representative western blots analysis of total protein from adult control and *Apc^Foxj1^* testes. β-actin was used as control. **C** Real-time PCR of Notch-target genes in control and *Apc^Foxj1^* testes at 6 month to 12-month-old. Values are fold induction or inhibition compared to c*ontrols* (arbitrarily adjusted to 1) (n = 3 for each genotype).

In the control testis from young mice, NICD was clearly present in the SSC, and differentiated spermatocytes and spermatids, suggesting active Notch signaling in the testicular epithelium (Fig. 10B&C). In the mutant testis, NICD was absent in SSC with Ctnnb1 accumulation (Fig. 10E&F). In older mutant mouse testes NICD was expressed only in few remaining germ cells (Fig. 11A). Consistent with reduced Notch signaling, western blot analysis showed decreased NICD (Fig. 11B) and quantitative RT-PCR revealed decreased transcripts of its downstream targets *Hes* and *Hey* genes (Fig. 11C). Jag1 was also undetectable in the SSCs with accumulated Ctnnb1 (Fig. S8). Thus in the testes, inactivation of *Apc* and consequent hyperactivation of Wnt signaling inhibit (rather than increase) Notch signaling, which is opposite to the observation in the lung. Thus inhibition of Notch signaling in the SSC may underlie their failure to maintain a reservoir population of stem cells.

## Discussion

Using *Foxj1-cre*, we inactivated *Apc* by targeted deletion of exon 14 specifically in cells committed to differentiate along ciliated cell lineage in various organs, including the lungs and the testes, two tissues analyzed in detail in this study. Our results reveal that in the lung, disruption of *Apc* activated the Wnt/Ctnnb1 signaling which in turn induced Notch and inhibited differentiation of only a sub-population of ciliated cell progenitors in the bronchiolar epithelium. In contrast, absence of *Apc* in the testes silenced Notch and caused male infertility by depletion of spermatogonial stem cells (SSC).

In the lung, *Apc* inactivation blocked only the differentiation of approximately 30% of the bronchiolar epithelial cells with evidence of commitment to ciliated cell program (*Foxj1-cre^positive^*). This suggests that the latter cells are derived from a progenitor pool distinct from that which gives rise to all other ciliated cells along the tracheal, bronchial and parts of the bronchiolar epithelia. Ciliated cells are thought to arise from two sources. The embryonic lung endoderm serves as a major source of progenitor cells. Alternatively, they can be generated by “trans-differentiation” from “facultative” Clara-like progenitors [28], [29], [30]. We propose that it is the latter pool of progenitors that fails to undergo trans-differentiation thus accounting for the approximately 30% Ctnnb1^pos^; βIV-tub^neg^ cells in the bronchiolar epithelium. Although this proposed scheme remains to be experimentally validated by cell lineage analysis, it is consistent with the finding by Reynold’s et al that stabilization of Ctnnb1 blocks Clara to ciliated cell differentiation in the bronchiolar epithelium, increases the number of naphthalene-resistant reparative cell pool and depletes the ciliated cell population [31].

Are facultative Clara-like cells in the mutant lung different compared to their wild type counterparts? In *Apc^Foxj1^* lungs, inactivation of Apc which potentiated Wnt signaling in ciliated cells via Ctnnb1 (Foxj1^pos^) also induced Jag1, which in turn uniformly induced Notch signaling in all neighboring Clara cells via juxtacrine activation. These must include also the “facultative” Clara cells (Please see below). Jag1, a known Notch ligand is a target of Wnt signaling [32] and its expression pattern in the embryonic mouse lung has been elucidated [20]. Thus, unlike the endodermal progenitors, in which Ctnnb1 and Wnt signaling predominate, in facultative Clara cells Ctnnb1 is activated in an environment that is already enriched in Notch signaling (Fig. 3). We speculate that increased Notch signaling favors maintenance of a stem cell status in Clara facultative cells and opposes ciliated cell trans-differentiation, hence increased Clara cell genes expression and decreased βIV-tub^pos^ cell numbers in *Apc^Foxj1^* lungs (Fig. 2). The above speculation is consistent with a number of findings. Notch is required for Clara cell regeneration and its inactivation expands ciliated cell populations, suggesting that Notch normally limits ciliated cell differentiation [18], [20]. Furthermore, in human airway epithelium and *Xenopus* laevis embryonic epidermis Notch signaling must undergo miR-449 microRNA inhibition to permit ciliated cell differentiation [33]. Thus, blocked differentiation of ciliated cells in the bronchiolar epithelium may be attributed to the functional outcome of time- and likely magnitude-dependent interplay between Wnt/Ctnnb1 and Notch signaling pathways.

Specific evidence that Notch is increased in all Clara cells including the “facultative” Clara or progenitor/stem cells was provided by the Naphthalene injury study. Recently, we reported that Notch1 is required for regeneration of Clara cells during repair of Naphthalene-induced airway injury [18]. Exposure of the bronchiolar epithelium to Naphthalene results in severe and rapid loss of the majority of Clara cells within 24 hours [5]. A select but small subpopulation of “facultative” Clara cells are resistant to Naphthalene and are thought to act as progenitors during regeneration of the bronchiolar epithelium [23], [24]. If the population of facultative progenitor cells with high Notch1 activity is indeed increased in *Apc^Foxj1^* lungs, we predicted measurable differences between the mutant and control mice in the timing of Clara cell regeneration & repair after Naphthalene-induced injury. In fact a clearly more accelerated repair of the airway epithelium was detected in the mutant lungs (Fig. 4). We propose that an increase in the sub-population of Clara cell progenitors in *Apc^Foxj1^*lungs, due to increased Notch signaling facilitates the repair of the naphthalene-injured airways. Thus, although we found blocked trans-differentiation of the facultative Clara cells to bronchiolar ciliated cells in *Apc^foxj1^* lungs, their increased pool nevertheless retained the ability to serve as progenitors for Clara cell differentiation/restoration in response to injury.

In this study, we provide the first direct evidence for a function of Apc as a key regulator of spermatogenesis. *Apc* inactivation caused male infertility. In wild type mice, a complex set of step-wise differentiation events leads from spermatogonia to spermatids and finally mature spermatozoa [34]. Spermatogonia serve as stem cells by undergoing asymmetrical cell division that serves to self-renew, and provide a continuous source of progenitors for production of mature sperm. In *Apc^Foxj1^* testes, spermatogonia are initially present at densities comparable to controls. However, there is a time-dependent progressive depletion of their pools in the mutant mice. Examination of proliferation revealed reduced mitotic index for the mutant SSCs (Fig. 9). Thus, inactivation of *Apc* and hyperactivation of Wnt/Ctnnb1 signaling interrupts what appears to be asymmetrical cell division that maintains a pool of SSCs throughout the life of male mice.

Importantly, and in contrast the lung, inactivation of *Apc* and accumulation of Ctnnb1 silenced Notch signaling in the SSC. Whether silencing of Notch is a direct impact of *Apc* inactivation or increased Wnt/Ctnnb signaling remains unknown. Nevertheless, silencing Notch may explain progressive loss of SSC as it is known to be necessary for maintenance of stem cells in many organs [35] and germ cell development in *Caenorhabditis elegans* [36] and *Drosophila* [37], [38]. Apc may also directly regulate SSC asymmetrical cell division by direct impact via its role as a mitotic spindle checkpoint [39]. Apc forms complexes with other proteins including Bub1 during mitosis [40]. Inactivation of *Bub1* in adult males inhibits spermatogonial proliferation [41]. In *Drosophila,* male germline stem cells are dependent on Apc to orient mitotic spindles perpendicular to the niche, ensuring a reliably asymmetric cell division [42]. Thus, this important function of Apc in maintenance of stem cell pools appears to be conserved over nearly five hundred million years of evolution.

Interestingly, the effect of *Apc* deletion on Notch signaling is different between lung and testis. In the lung, ectopic Wnt signaling is reported to attenuate airway cell differentiation [31]. This is not what we observed in *Apc^Foxj1^* lungs. We believe this is primarily due to the timing of *Foxj1-cre* activation, which occurs subsequent to cell fate commitment in the majority of endodermally derived ciliated cell progenitors. Thus, in the proximal conducting airways of the mutant lung, differentiated ciliated cell numbers were wild-type like. The exception was blocked differentiation of a subset of *Foxj1^pos^* cells in the bronchiolar epithelium. These cells had already committed to differentiate along ciliated cell phenotype, but had not proceeded to overt differentiation (expression of βIV-tubulin). We propose that such cells originate from facultative Clara cells whose molecular phenotype in the mutant lung is altered by induction of Notch signaling. In the lung and in particular the facultative Clara cells, activation of Notch which occurs via lateral induction of Jag1 temporally preceded potentiation of Wnt signaling (increased Ctnnb1) triggered by Foxj1-cre-mediated recombination. Notch is well recognized to favor maintenance of pluripotency and its interruption favors ciliated cell hyperplasia [18], [20]. Compared to the lung, we found a significantly different Wnt∼Notch signaling environment in the testis. We propose that this is principally due to timing of Foxj1 activation which in contrast to the lung occurs early in spermatogonial stem cells. This leads to early activation of Wnt signaling by stabilized Ctnnb1, which is a known inhibitor of Notch signaling [43]. Here gradual depletion of spermatogonial stem cell pools was observed. In addition, Jag1 whose expression triggered increased Notch signaling in the lung is not expressed in the testis [44] (Fig. S8). Thus, in the testis, Wnt inhibits Notch activity to promote a Wnt-ON/Notch-OFF output, thereby enhancing the differentiation rather than “stem-cell-ness” of spermatogonial stem cells, which eventually caused depletion of the SSC reservoir (Fig. 9). However, we cannot exclude the possibility that given the multifunctional properties of Apc, disruption of its functions other than Wnt∼Notch signaling may also have contributed to the observed overt phenotype in the lung and testis. Apc is well known to play a role in regulating junction structure and function in the intestine [45] and testis [46]. Although histologically we observed no gross or fundamental epithelial abnormalities in *Apc^Foxj1^* mutant lungs, the nuclear Ctnnb1 accumulation in the spermatogonia could cause defects in sertoli-spermatogonia adhesion located in the basal compartment of blood-testis barrier, which may ultimately lead to germ cell loss. Further studies are needed to determine what role if any Notch may play in maintaining integrity of testicular epithelium.

Infertility in men is widespread yet poorly understood. Failure in spermatogenesis appears frequently as idiopathic and may be due to genetic causes [47]. Apc is expressed in some spermatogonia and interstitial (Leydig) cells of human fetal testis [48] and in germ cells and Leydig cells of the adult [49]. This is in agreement with our IHC results in mice where Apc is highly expressed in spermatogonia with lower expression found in other cells of the seminiferous tubules (Fig. 7) suggesting a role for *Apc* in testicular development and maintenance. Extrapolation based on the similarities between mouse and human *Apc* expression patterns raises the possibility that mutations in the human *Apc* gene may be contributory to infertility in man.

Finally, we provide evidence that *Foxj1* expression is directly activated by Apc (Fig. 5C and Fig. S9). As this work was in progress, another Wnt/Ctnnb1 signaling negative regulator, Chibby *(*Cby1*)* was identified as a regulator of ciliogenesis [50]. Of interest, while *Cby1* is directly activated by *Foxj1* in the latter study, we found that both genes are affected by Apc (Fig. S9). Compelling evidence has been obtained by studying a zebrafish Foxj1 protein that the principal mechanism by which Foxj1 controls motile ciliogenesis is through the transcriptional regulation of genes encoding components that are critical for the synthesis and function of motile cilia [9]. Our in vitro data demonstrate that by regulating Jag1 and consequently Notch signaling in the airway epithelium, Apc can control the fate of a select group of progenitor cells that give rise to the bronchiolar epithelium. The bronchiolar epithelium is currently thought to be the site of important facultative stem cells. For example, bronchoioalveolar stem cells (BASCs), distinguished by Spc/CC10 double expression are thought to reside within this region at the cross section between conducting airways and the respiratory compartment of the lung [51]. The observation that *Foxj1-cre* mediated inactivation of *Apc* does not block ciliated cell differentiation throughout the airway epithelium suggests functional differences between the progenitors from which the proximal versus bronchiolar ciliated cells are derived. In this regard the timing of Apc inactivation, dictated by the specific promoter that drives cre expression is an important factor in determining the outcome. For example, in *Nkx2.1-cre; Apc^flox/folx^* lungs, differentiation of both ciliated and Clara cells throughout the airway epithelium is blocked (Li et al., Submitted).

In summary, the current study reveals the important role of Apc as a key player in the processes of ciliogenesis and spermatogenesis in vivo. To date, no mutations in the human *Apc* have been identified that result in male sterility. However, given the cilia and flagella phenotypes uncovered in *Apc^Foxj1^* mice by the present study, elucidating the function of Apc may contribute greatly to the understanding of the molecular mechanisms perturbed in human cilia-related disorders including PCD, and may aid in their diagnosis and treatment.

## Supporting Information

Figure S1 **Foxj1-cre expression pattern during murine lung development.** **A–C** LacZ staining of cre-induced β-galactosidase (β-gal) activity in whole mount lungs of *Foxj1-cre; Rosa26R* mouse. (A) Embryonic day 16 (E16) lungs. No LacZ staining was seen. (B) E18 lungs, *Foxj1-cre* mediated recombination was visible. (C) Postnatal day 3 (PN3) lungs, LacZ is similar to E18. **D–F** IHC for β-gal in *Foxj1-cre; Rosa26R* E16 lungs, counterstained with methyl green. β–gal signaling (brown) was localized to epithelial cells (arrows) of trachea (D), bronchi (E) and bronchioles (F). **G–I** LacZ staining of *Foxj1-cre;Rosa26R* PN3 lungs. LacZ staining (blue) was localized to epithelial cells (arrows) of trachea (G), bronchi (H) and bronchioles (I). Scale bar: 2 mm for Panels A–C; 40 µm for Panels D–I.
(TIF)

Figure S2 **Foxj1-cre expression pattern in the adult** ***Foxj1-cre; mTmG*** **mouse testis. A** Whole mount testis in red fluorescence showing absent recombination. **B** Green fluorescence showing *Foxj1-cre* positive recombination. **C** Testis section showing strong green fluorescence signal located in spermatogonia, spermatids and spermatozoa (arrows) of semiferous epithelium. Scale bars: 2 mm for A&B 100 µm for C.
(TIF)

Figure S3 **Wnt/Ctnnb1 signaling is activated in Ctnnb1 accumulated cells. A&E** X-gal staining of control (*Apcflox/flox; Aixn2-LacZ*) and *Apc^Foxj^ (Foxj1-cre; Apcflox/flox; Aixn2-LacZ) lungs* at postnatal 2 week-old mice. Arrows indicate two epithelial cells with strong LacZ activity. **B–H** Immunostaining of β-gal (red) and Ctnnb1 (green) on the X-gal stained sections. (**B–D**) Control lung. (**F–H**) Mutant lung. Arrows in **F, G** & **H** show X-gal stained epithelial cells are both β-gal^pos^ and Ctnnb1^pos^. Scale bar: 40 µm.
(TIF)

Figure S4 **Wnt/Ctnnb1 signaling is active only in Ctnnb1^pos^ cells.** Immunostaining of Ctnnb1 or CC10 (green) and Axin2 (red) in control (**A&C**) and *Apc^Foxj1^* (B&D) lungs. Arrows in B show co-localization of Ctnnb1 with the Wnt-target gene, Axin2. Arrows in D show absence of co-localization of CC10 with Axin2. Scale bar: 10 µm.
(TIF)

Figure S5 **Loss of** ***Apc*** **does not affect cell proliferation. A** Representative immunostaining of Ctnnb1 (green) and Ki67 (red) in control and *Apc^Foxj1^* lungs. Arrows in F show Ki67^pos^ cells; asterisks show cells with accumulated Ctnnb1. Note: the cells with accumulated Ctnnb1 are not Ki67^pos^. Scale bar: 20 µm. **B** Quantification of Ki67^pos^ cells by manual counting in control and *Apc^Foxj1^* lung from 2-weeks to adult (n = 3 for each genotype).
(TIF)

Figure S6 **Wnt/Ctnnb1 signaling is active in the Ctnnb1accumulated spermatogonia. A** Whole mount X-gal staining of control (*Apcflox/flox; Axin2-LacZ,* left) and *Apc^Foxj1^* mutant (*Foxj1-cre; Apcflox/flox; Aixn2-LacZ*, right) testes. Note robust dark staining of β-gal (LacZ) in the *Apc^Foxj1^* testis (right) although the staining is too dark to see the detail. Scale bar: 2 mm. **B & C** Immunostaining of Axin2 (red) and Ctnnb1 (green) in control (B) and mutant (C) testes. Arrows in C show co-localization of Ctnnb1 with Axin2. Scale bar: 10 µm.
(TIF)

Figure S7 **Co-localization of Apc and PLZF in wild-type mouse testes.** Immunostaining of Apc (green) and PLZF (red) in postnatal 3 days and 2 month testes of wild type mice. Arrows indicate co-localization of Apc and PLZF signals. Dotted lines indicate the basement membrane of seminiferous tubules. Scale bar: 20 µm.
(TIF)

Figure S8 **Inactivation of Notch pathway in the** ***Apc^Foxj1^*** **mouse testis.** Immunostaining of Ctmmb1 (green) and Jag1 (red) in control (A) and *Apc^Foxj1^* testes (B). Arrows in B indicate Ctnnb1^pos^ spermatogonia are Jag1^neg^. Scale bar: 30 µm.
(TIF)

Figure S9 **Loss of** ***Apc*** **decreased motile ciliogenic gene expression in the** ***Apc^Foxj1^*** **mouse lung and testis. A** Real-time PCR of *Foxj1* mRNA level in control and *Apc^Foxj1^* lungs and testes. **B** Real-time PCR of *Cby1* mRNA level in control and *Apc^Foxj1^* lungs and testes. Values are fold inhibition compared to *controls* (arbitrarily adjusted to 1) and mean ± SD are shown (n = 3 for each genotype).
(TIF)

Table S1 **Primary antibodies used in western blots or immunohistochemistry.**
(DOC)
