# Correction: Tissue-Dependent Consequences of *Apc* Inactivation on Proliferation and Differentiation of Ciliated Cell Progenitors via Wnt and Notch Signaling

**DOI:** 10.1371/annotation/79fc47f5-11a5-4ef3-a80e-e2c5af62c5c1

**Published:** 2013-05-30

**Authors:** Aimin Li, Belinda Chan, Juan C. Felix, Yiming Xing, Min Li, Steven L. Brody, Yong Zhang, Michael J. Holtzman, Zea Borok, Changgong Li, Parviz Minoo

There were author omitted from the author list. The correct list and associated informations are available below.

Aimin Li^1^, Belinda Chan^1^, Juan C. Felix^2^, Yiming Xing^1,5^, Min Li^1^, Steven L. Brody^3^, Yong Zhang^3^, Michael J. Holtzman^3^, Zea Borok^4^, Changgong Li^1^ and Parviz Minoo^1*^

1. Department of Pediatrics, Division of Newborn Medicine, Los Angeles County+University of Southern California Medical Center, Keck School of Medicine of USC, Los Angeles, CA90033

2. Department of Pathology, Los Angeles County+University of Southern California Medical Center, Los Angeles, CA90033

3. Pulmonary and Critical Care Medicine, Department of Medicine, Washington University School of Medicine, St. Louis, MO63110.

4. Will Rogers Institute Pulmonary Research Center, Division of Pulmonary and Critical Care Medicine, Department of Medicine, and Department of Biochemistry and Molecular Biology, University of Southern California, Keck School of Medicine of USC, Los Angeles, CA 90033

5. The State Key Laboratory for Agro-biotechnology, China Agricultural University, Beijing 100094, China

Author Contributions: Conceived and designed the experiments: PM AL. Performed the experiments: AL BC ML YX. Analyzed the data: PM AL CL ZB SLB JCF. Contributed reagents/materials/analysis tools: YZ MJH. Wrote the paper: PM AL. 

