# Supplementary figures and images for: Tissue-Dependent Consequences of Apc Inactivation on Proliferation and Differentiation of Ciliated Cell Progenitors via Wnt and Notch Signaling
Source: PLoS One. 2013 Apr 30;8(4):e62215. doi: 10.1371/journal.pone.0062215 (PMC3639955; doi:10.1371/journal.pone.0062215)

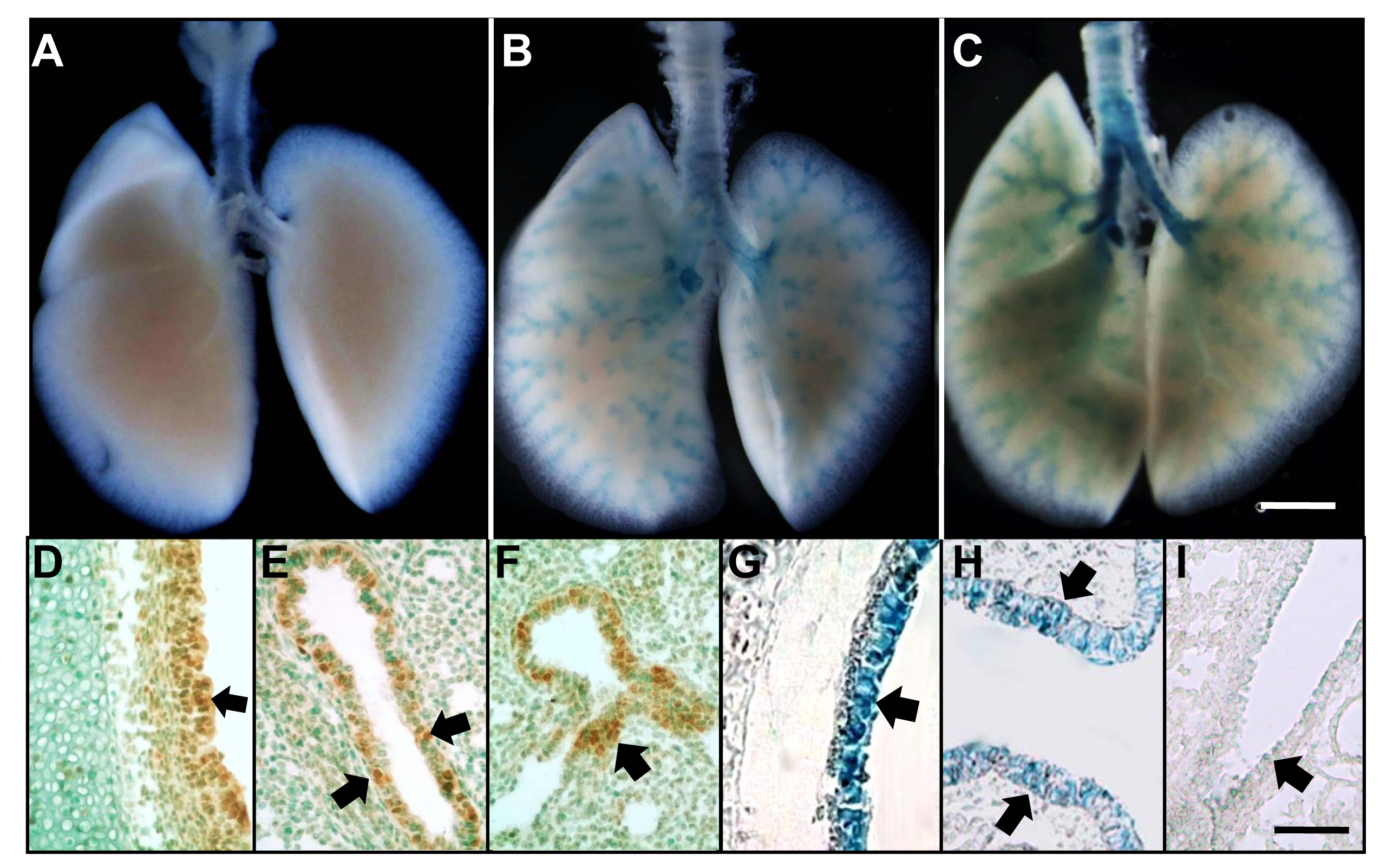

Supplement: Figure S1 — Foxj1-cre expression pattern during murine lung development. A–C LacZ staining of cre-induced β-galactosidase (β-gal) activity in whole mount lungs of Foxj1-cre; Rosa26R mouse. (A) Embryonic day 16 (E16) lungs. No LacZ staining was seen. (B) E18 lungs, Foxj1-cre mediated recombination was visible. (C) Postnatal day 3 (PN3) lungs, LacZ is similar to E18. D–F IHC for β-gal in Foxj1-cre; Rosa26R E16 lungs, counterstained with methyl green. β–gal signaling (brown) was localized to epithelial cells (arrows) of trachea (D), bronchi (E) and bronchioles (F). G–I LacZ staining of Foxj1-cre;Rosa26R PN3 lungs. LacZ staining (blue) was localized to epithelial cells (arrows) of trachea (G), bronchi (H) and bronchioles (I). Scale bar: 2 mm for Panels A–C; 40 µm for Panels D–I. (TIF) [file pone.0062215.s001.tif]

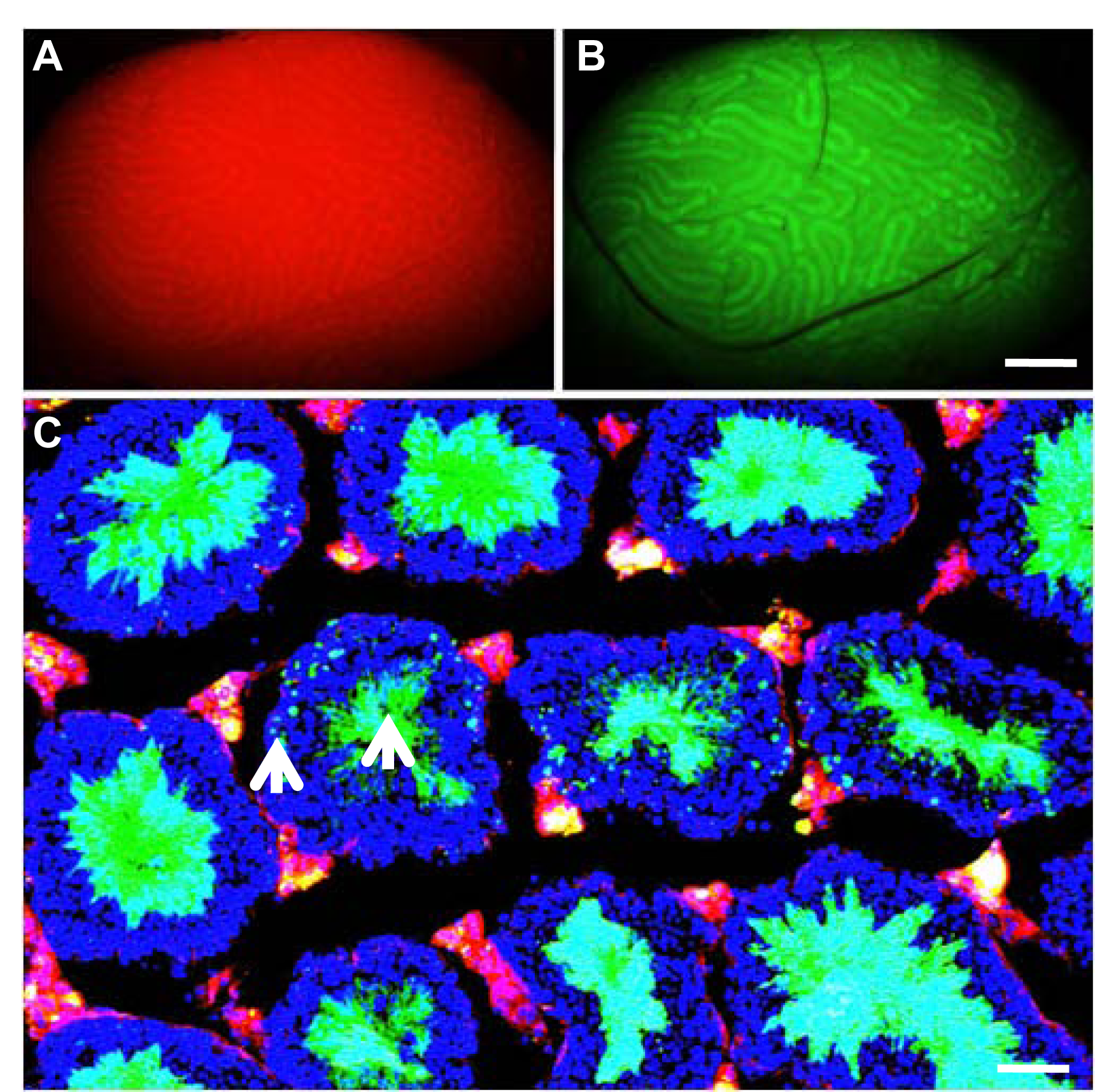

Supplement: Figure S2 — Foxj1-cre expression pattern in the adult Foxj1-cre; mTmG mouse testis. A Whole mount testis in red fluorescence showing absent recombination. B Green fluorescence showing Foxj1-cre positive recombination. C Testis section showing strong green fluorescence signal located in spermatogonia, spermatids and spermatozoa (arrows) of semiferous epithelium. Scale bars: 2 mm for A&B 100 µm for C. (TIF) [file pone.0062215.s002.tif]

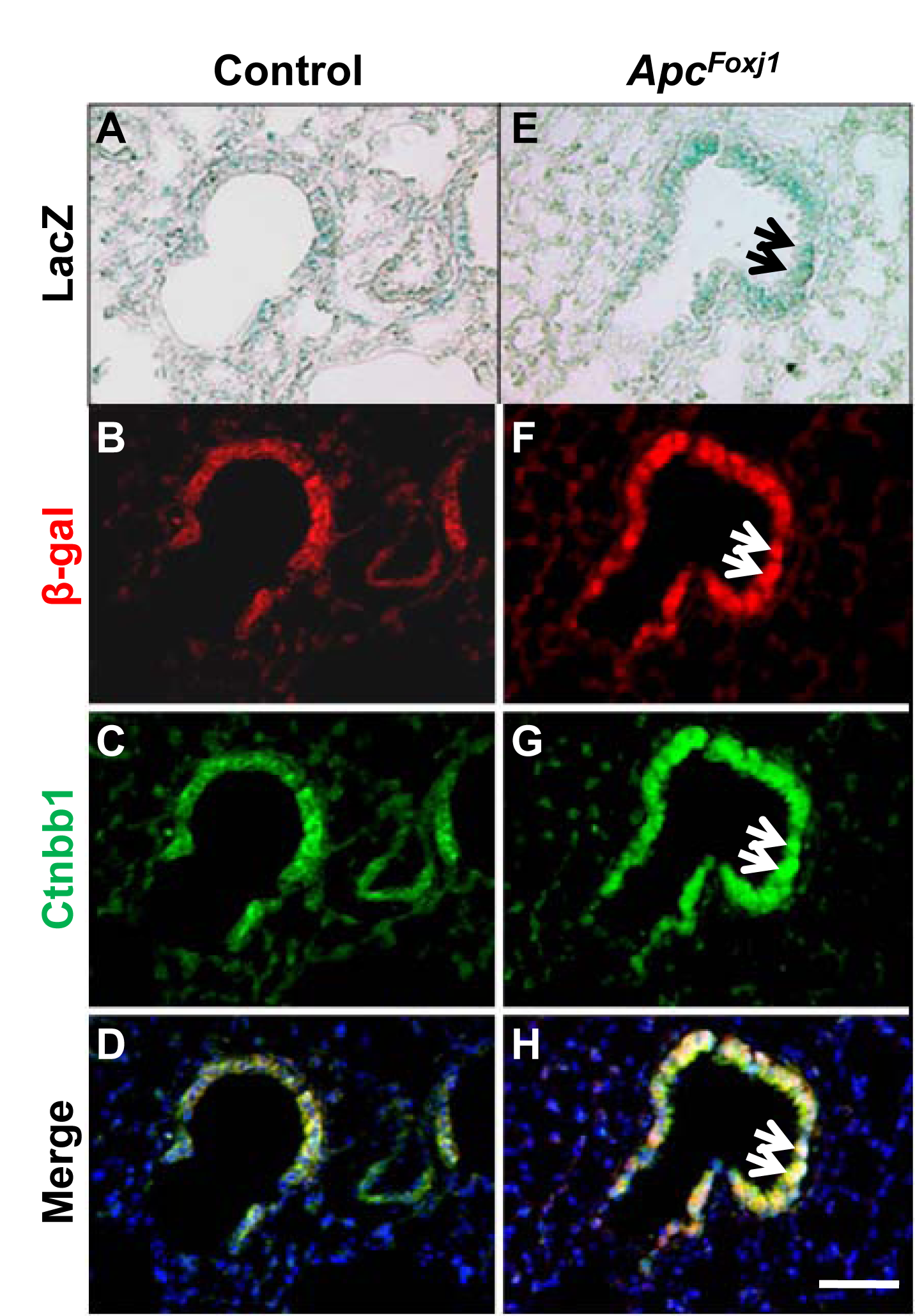

Supplement: Figure S3 — Wnt/Ctnnb1 signaling is activated in Ctnnb1 accumulated cells. A&E X-gal staining of control (Apcflox/flox; Aixn2-LacZ) and ApcFoxj (Foxj1-cre; Apcflox/flox; Aixn2-LacZ) lungs at postnatal 2 week-old mice. Arrows indicate two epithelial cells with strong LacZ activity. B–H Immunostaining of β-gal (red) and Ctnnb1 (green) on the X-gal stained sections. (B–D) Control lung. (F–H) Mutant lung. Arrows in F, G & H show X-gal stained epithelial cells are both β-galpos and Ctnnb1pos. Scale bar: 40 µm. (TIF) [file pone.0062215.s003.tif]

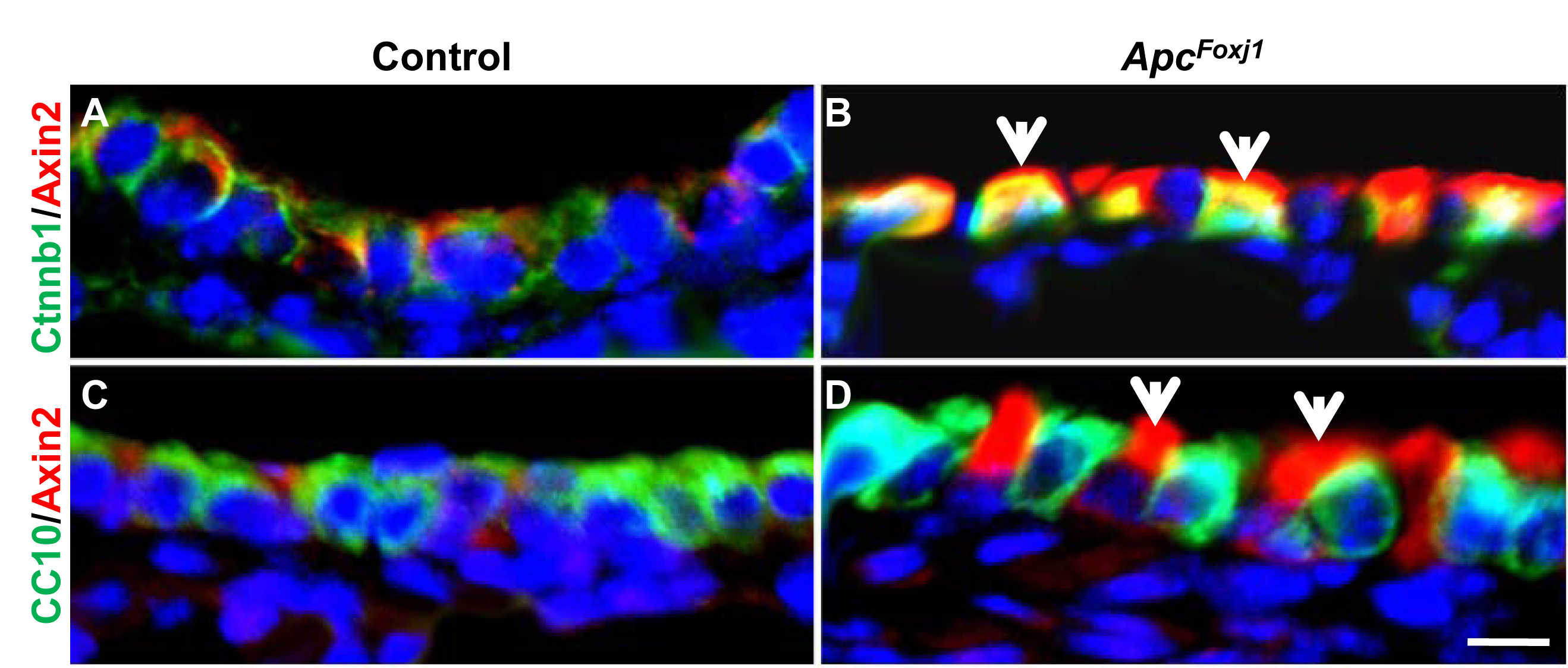

Supplement: Figure S4 — Wnt/Ctnnb1 signaling is active only in Ctnnb1pos cells. Immunostaining of Ctnnb1 or CC10 (green) and Axin2 (red) in control (A&C) and ApcFoxj1 (B&D) lungs. Arrows in B show co-localization of Ctnnb1 with the Wnt-target gene, Axin2. Arrows in D show absence of co-localization of CC10 with Axin2. Scale bar: 10 µm. (TIF) [file pone.0062215.s004.tif]

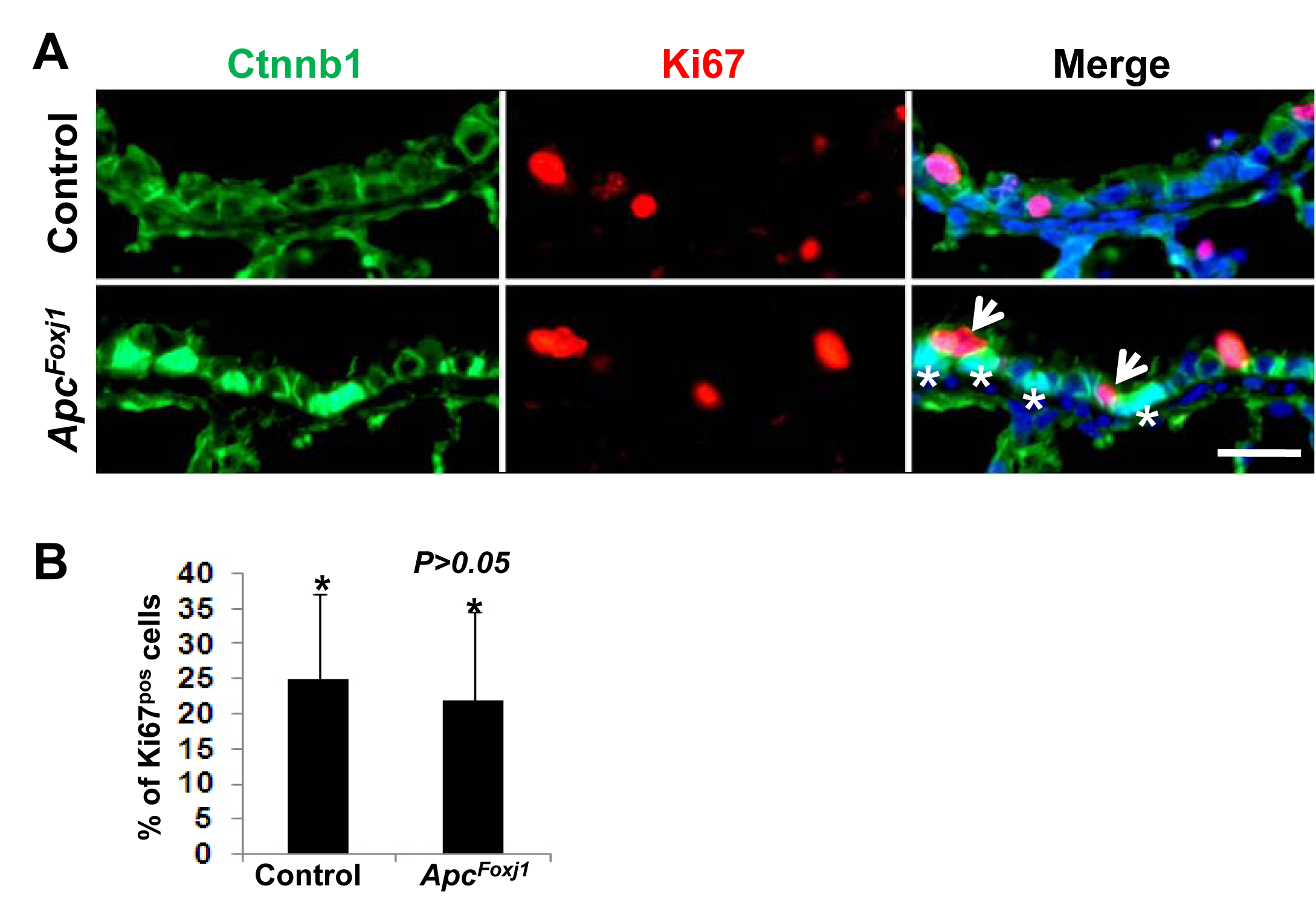

Supplement: Figure S5 — Loss of Apc does not affect cell proliferation. A Representative immunostaining of Ctnnb1 (green) and Ki67 (red) in control and ApcFoxj1 lungs. Arrows in F show Ki67pos cells; asterisks show cells with accumulated Ctnnb1. Note: the cells with accumulated Ctnnb1 are not Ki67pos. Scale bar: 20 µm. B Quantification of Ki67pos cells by manual counting in control and ApcFoxj1 lung from 2-weeks to adult (n = 3 for each genotype). (TIF) [file pone.0062215.s005.tif]

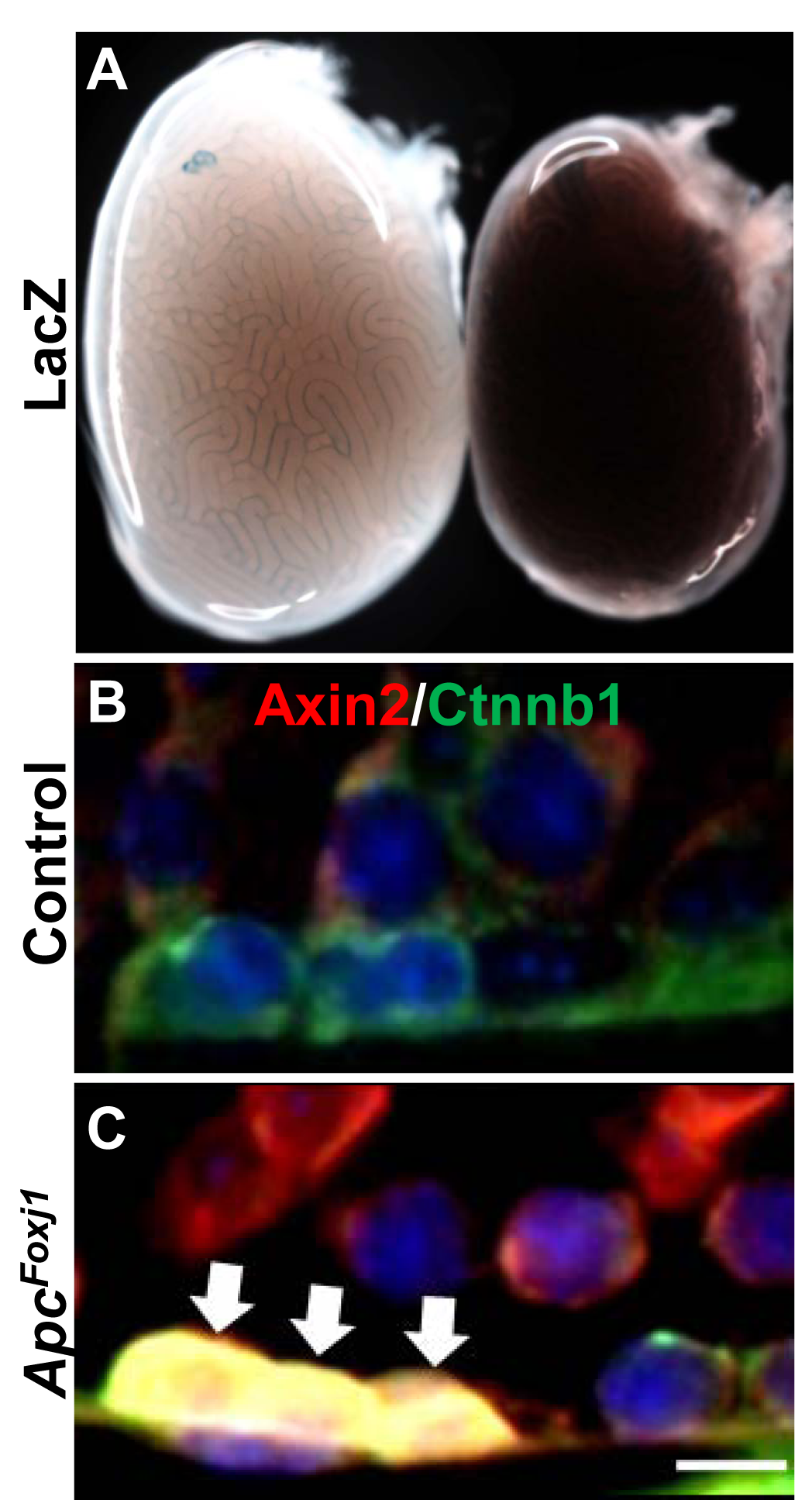

Supplement: Figure S6 — Wnt/Ctnnb1 signaling is active in the Ctnnb1accumulated spermatogonia. A Whole mount X-gal staining of control (Apcflox/flox; Axin2-LacZ, left) and ApcFoxj1 mutant (Foxj1-cre; Apcflox/flox; Aixn2-LacZ, right) testes. Note robust dark staining of β-gal (LacZ) in the ApcFoxj1 testis (right) although the staining is too dark to see the detail. Scale bar: 2 mm. B & C Immunostaining of Axin2 (red) and Ctnnb1 (green) in control (B) and mutant (C) testes. Arrows in C show co-localization of Ctnnb1 with Axin2. Scale bar: 10 µm. (TIF) [file pone.0062215.s006.tif]

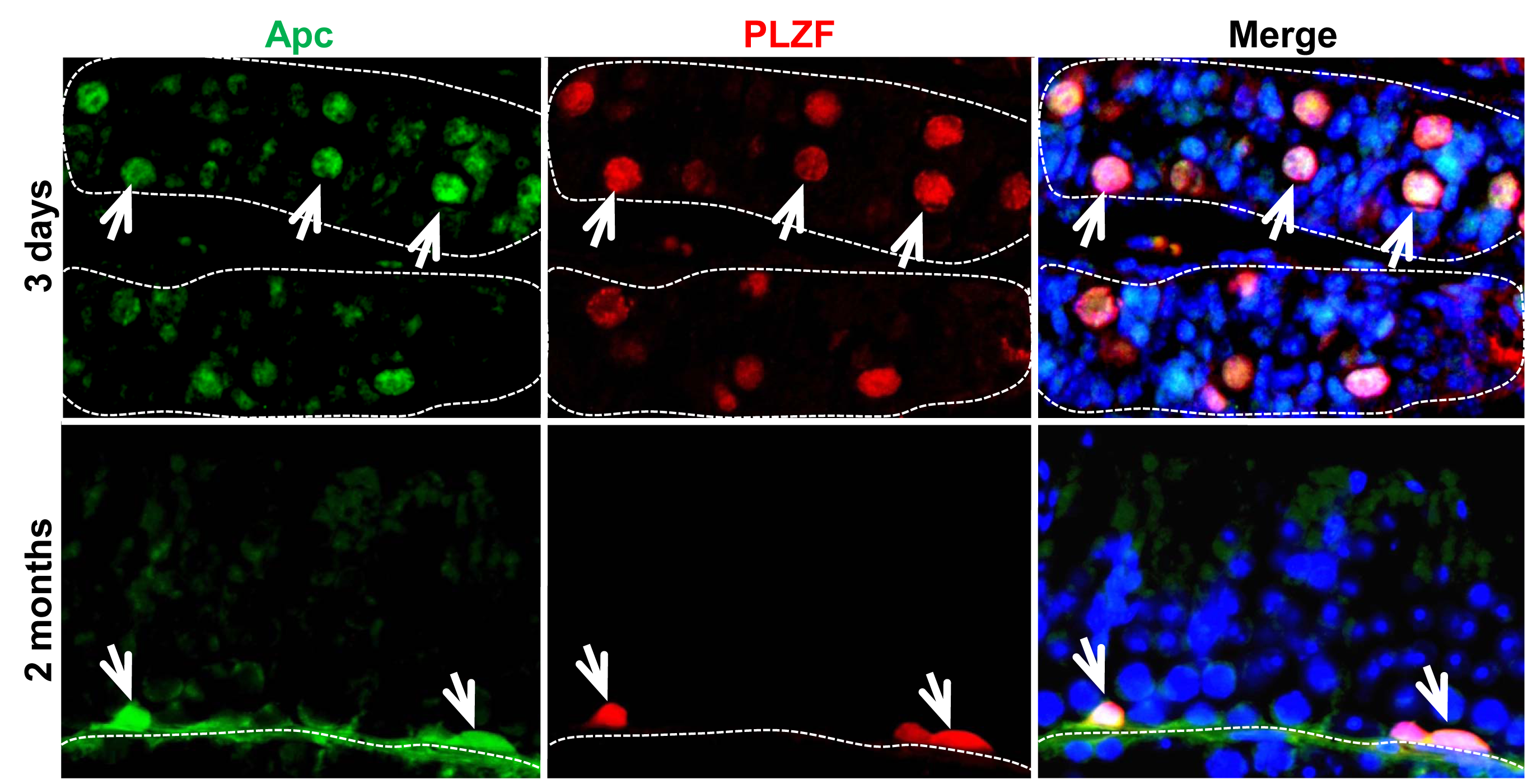

Supplement: Figure S7 — Co-localization of Apc and PLZF in wild-type mouse testes. Immunostaining of Apc (green) and PLZF (red) in postnatal 3 days and 2 month testes of wild type mice. Arrows indicate co-localization of Apc and PLZF signals. Dotted lines indicate the basement membrane of seminiferous tubules. Scale bar: 20 µm. (TIF) [file pone.0062215.s007.tif]

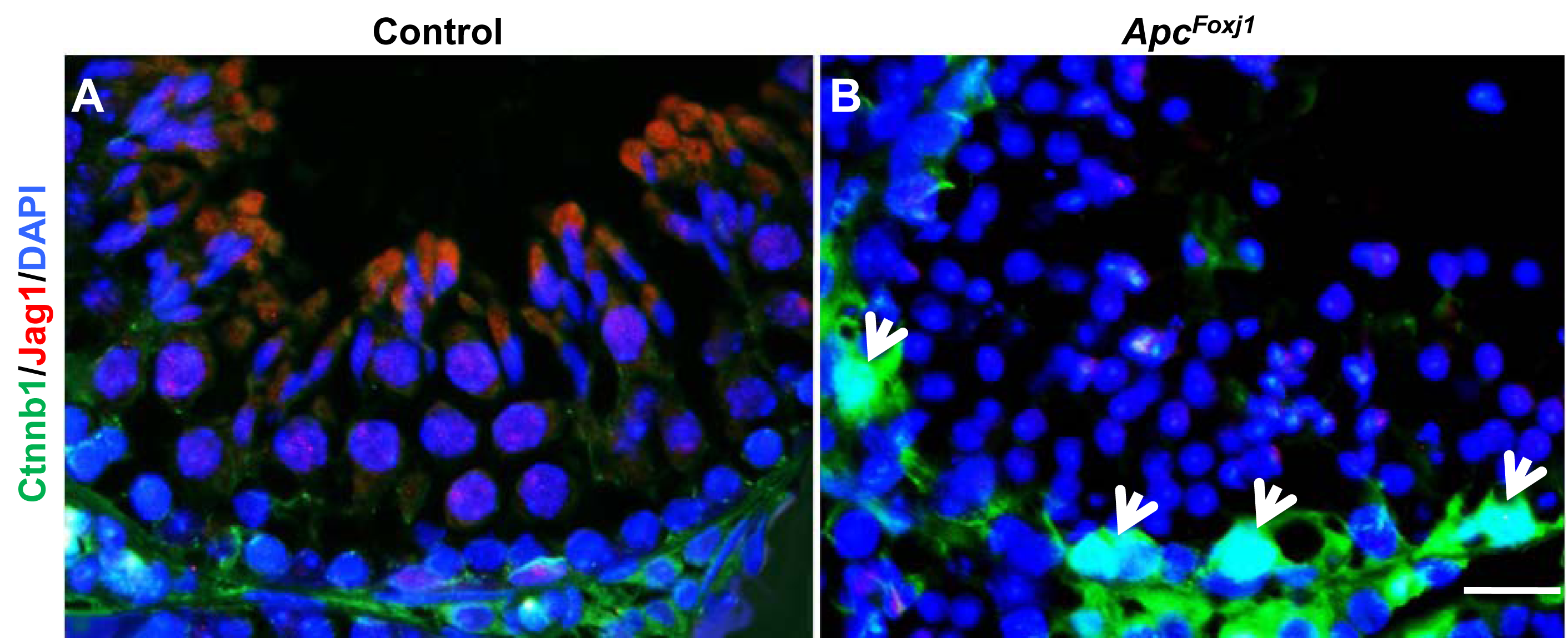

Supplement: Figure S8 — Inactivation of Notch pathway in the ApcFoxj1 mouse testis. Immunostaining of Ctmmb1 (green) and Jag1 (red) in control (A) and ApcFoxj1 testes (B). Arrows in B indicate Ctnnb1pos spermatogonia are Jag1neg. Scale bar: 30 µm. (TIF) [file pone.0062215.s008.tif]

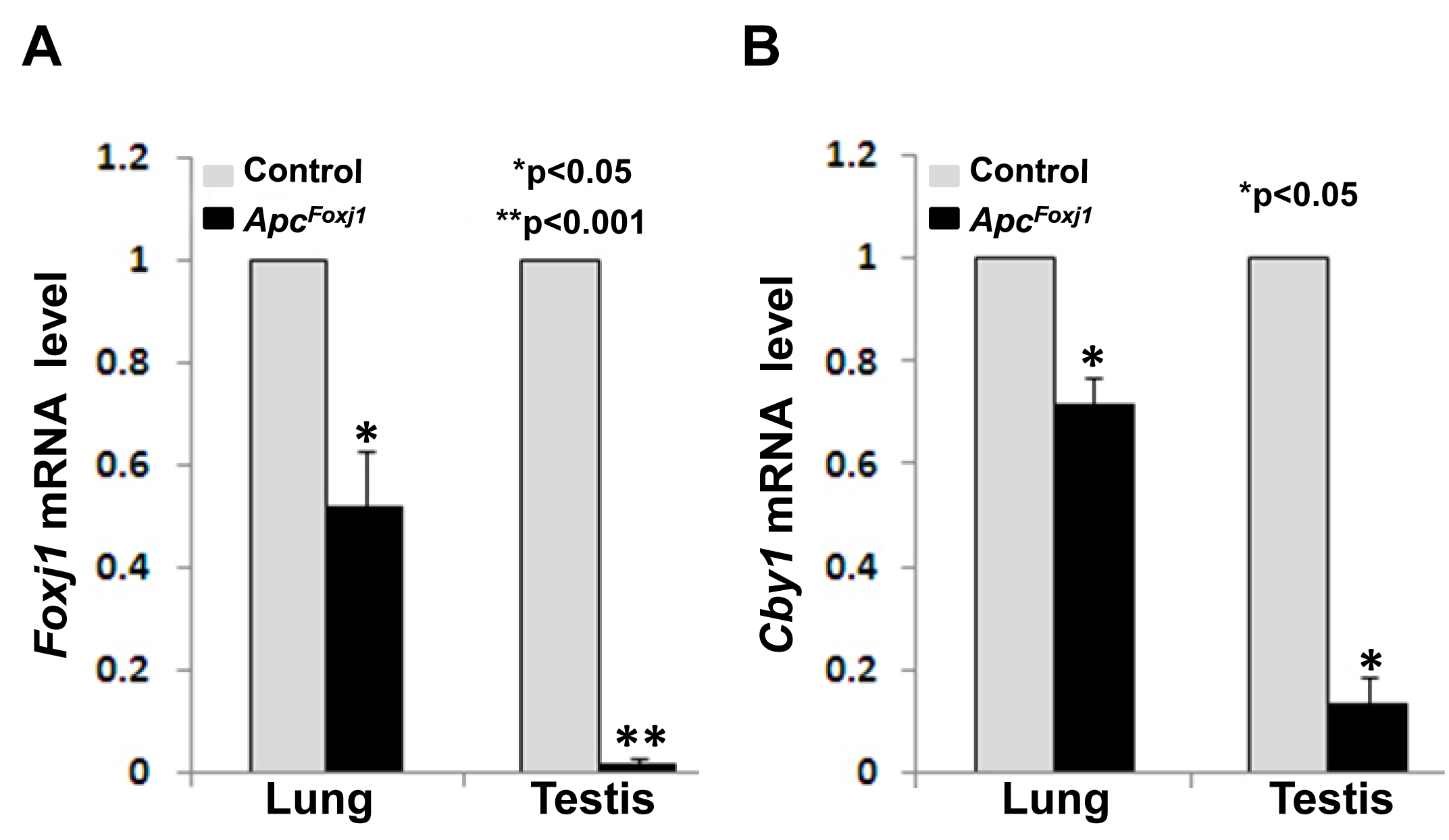

Supplement: Figure S9 — Loss of Apc decreased motile ciliogenic gene expression in the ApcFoxj1 mouse lung and testis. A Real-time PCR of Foxj1 mRNA level in control and ApcFoxj1 lungs and testes. B Real-time PCR of Cby1 mRNA level in control and ApcFoxj1 lungs and testes. Values are fold inhibition compared to controls (arbitrarily adjusted to 1) and mean ± SD are shown (n = 3 for each genotype). (TIF) [file pone.0062215.s009.tif]
