# Supplementary material for: Tissue-Dependent Consequences of Apc Inactivation on Proliferation and Differentiation of Ciliated Cell Progenitors via Wnt and Notch Signaling
Source: PLoS One. 2013 Apr 30;8(4):e62215. doi: 10.1371/journal.pone.0062215 (PMC3639955; doi:10.1371/journal.pone.0062215)
Supplement: Table S1 — Primary antibodies used in western blots or immunohistochemistry. (DOC) [file pone.0062215.s010.doc]

| **Table S1. Primary antibodies used in western blots or immunohistochemistry** | | |
| --- | --- | --- |
| **Name** | **Type** | **Source** |
| Apc | Rabbit polyclonal | Anaspec |
| Axin2 | Rabbit polyclonal | Abcam |
| α-tubulin | Mouse monoclonal | Sigma |
| β-catenin | Mouse monoclonal | BD Transduction Laboratories |
| β-galactosidase | Mouse monoclonal | Sigma |
| βIV-tubulin | Mouse monoclonal | Biogenex |
| β-actin | Rabbit polyclonal | Abcam |
| CC10 | Goat polyclonal | Santa Cruz |
| DAZL | Mouse monoclonal | Santa Cruz |
| DDX4 | Rabbit polyclonal | Abcam |
| Foxj1 | Mouse monoclonal | Santa Cruz |
| Jagged1 | Rabbit polyclonal | Abcam |
| Ki67 | Rabbit monoclonal | Lab vision |
| Active-Notch1 (NICD) | Rabbit polyclonal | Abcam |
| Phospho-Histone-H3 | Mouse monoclonal | Cell Signaling Technology |
| PLZF | Goat polyclonal | Santa Cruz |
